# In-Depth Multi-Assembler Venom-Gland Transcriptomics of Three Medically Important Colombian Snakes Highlights Diversity of Accessory, Low-Abundance Protein Families

**DOI:** 10.3390/toxins18030118

**Published:** 2026-02-25

**Authors:** Mónica Saldarriaga-Córdoba, Claudia Clavero-León, Paola Rey-Suárez, Vitelbina Núñez-Rangel, Sebastián Estrada-Gómez

**Affiliations:** 1 Centro de Investigación en Recursos Naturales y Sustentabilidad (CIRENYS), Universidad Bernardo O’Higgins, Santiago 8320000, Chile; sebastian.estrada@udea.edu.co; 2 Escuela de Medicina Veterinaria, Universidad Bernardo O’Higgins, Santiago 8320000, Chile; 3 Grupo de Investigación en Toxinología, Alternativas Terapéuticas y Alimentarias, Facultad de Ciencias, Farmacéuticas y Alimentarias, Universidad de Antioquia, Medellín 50010, Colombia; jessicarey@itm.edu.co (P.R.-S.); vitelbina.nunez@udea.edu.co (V.N.-R.); 4 Facultad de Ciencias Exactas y Aplicadas, Institución Universitaria ITM, Medellín 50010, Colombia; 5 Escuela de Microbiología, Universidad de Antioquia, Medellín 50010, Colombia

**Keywords:** *Bothrops asper*, *Crotalus durissus cumanensis*, *Micrurus mipartitus*, transcriptomic analysis, de novo assembly, comparative transcriptomic, transcript diversity

## Abstract

Typically, most omics analysis (proteomic and transcriptomic) of snakes are focused on the dominant enzymatic proteins used for evolutionary analysis or those engaged in envenoming symptoms. This study presents a comprehensive multi-assembler transcriptomic analysis focused on the non-dominant and enzymatic or non-enzymatic putative proteins of the venom glands of three medically significant Colombian snake species. Together, these results highlight how continued improvements in modern omics workflows, coupled with extensive manual curation, enable more complete putative protein variants discovery when multiple assemblers are integrated. Here, we reconstructed the toxinomes of the viperids *Bothrops asper* and *Crotalus durissus cumanensis,* and the elapid *Micrurus mipartitus*, by comparing four assemblers (Trinity, SPAdes, SOAPdenovo-Trans k = 31 and k = 97) and integrating them into a non-redundant meta-assembly. Protein-candidate alignments were extensively inspected, and validation of conserved domains and functional motifs are discussed. The curated toxinomes revealed substantial diversity across major and accessory families, and assembler choice strongly affected transcript variant recovery. Together, these results provide a more comprehensive view of venom-gland transcriptome analysis and diversity, expanding the set of candidate venom components for future functional and proteomic validation, with potential implications for venom composition studies and antivenom development.

## 1. Introduction

Snake venom (SV) composition usually exhibits marked qualitative and quantitative variation across genera and species, and even among populations of the same species [1]. Omics approaches have been proven to be extremely effective for discovering and characterizing protein molecules, from low- to high-molecular-mass compounds (LMMCs–HMMCs). SV proteomics provides a remarkable snapshot of venom composition at a given time, which may vary according to the individual’s age, sex and biogeographic origin, whereas venom gland (VG) transcriptomics is one of the most powerful approaches to depict the broader landscape of venom composition, including both secreted and non-secreted proteins. In-depth analyses of venom-gland transcriptomes that combine multiple de novo assemblers and manual curation can reconstruct a more accurate and complete catalogue of protein-like genes (enzymatic or non-enzymatic genes), recovering distinct copies of enzymatic HMMCs and revealing venom variability across biological levels (within individuals, among populations and among species).

At the genomic level, the origin and diversification of SV toxins have been explained by at least two recruitment mechanisms. A hijacking/modification mechanism involves the direct specialization of ancestral non-toxin genes that were already present at the locus and expressed in other tissues, which become co-opted and transcriptionally specialized genes in the venom gland [2]. In parallel, a duplication and neofunctionalization mechanism, in which ancestral genes are duplicated and some of the copies accumulate structural and regulatory changes, allows the emergence of novel enzymatic proteins transcripts with modified biochemical activities and target specificities (toxins) [3]. Together, these recruitment mechanisms generate an extensive protein diversity and underlie the expansion of multigene protein families in venomous snakes, particularly within low-abundance gene copies that may represent recent or lineage-specific recruitment events and contribute to the accessory venom repertoire [4].

Snake VGs sustain a complex genomic and regulatory architecture that underlies the expression and secretion of a multiplex mixture of active peptides, proteins and other compounds, and are widely recognized as an important source of bioactive molecules. These SV proteins are often grouped into three major categories of HMMCs with complex molecular architectures: (i) enzymatic proteins (toxins), (ii) non-enzymatic proteins with regulatory or structural functions and (iii) proteins (non-toxin) with housekeeping cellular functions that reflect the intense secretory and metabolic activity of the tissue [5]. Enzymatic proteins correspond to the classical dominant and widely described toxins, including phospholipases A_2_ (PLA_2_), snake venom metalloproteinases (SVMP), snake venom serine proteinases (SVSP), myotoxins (MYO), Hyaluronidase (HYAL), Phospholipase B (PLB), Lipase (LIPA) and L-amino-acid oxidases (LAO) [6]. Non-enzymatic proteins comprise cardiotoxins, cytotoxins, C-type lectins/snaclecs (SNACLEC), three-finger toxins (3FTx), Kunitz-type protease inhibitors (KUN), disintegrins, acetylcholinesterase (AChE), cysteine-rich secretory proteins (CRISP), waprins (WAP), ficolins, cystatins and growth factors (e.g., vascular endothelial growth factor, VEGF; nerve growth factor, NGF) [6]. The third group consists of proteins such as disulfide isomerases, molecular chaperones and other endoplasmic reticulum and Golgi-resident proteins that are co-expressed with venom toxins and support the intense secretory activity of the venom gland. These proteins are involved in fundamental cellular process such as protein folding, post-translational modification and signaling pathway activation, and are usually reported at low abundance in venom transcriptome and proteome datasets [7,8,9,10]. Enzymatic and non-enzymatic venom proteins constitute the core toxin repertoire of snake venoms involved in envenoming manifestations. In contrast, while transcripts encoding proteins with housekeeping cellular functions are not considered part of the venom arsenal and do not contribute directly to envenoming, their expression provides important context on the cellular machinery that supports toxin synthesis, processing and export, and needs to be distinguished from venom-associated, accessory toxin families when interpreting venom-gland transcriptomic data [4].

Most venom-omics studies have primarily focused on dominant enzymatic proteins families (toxins), which account for the largest proportion of the proteome and are directly linked to prey immobilization, digestion and clinical manifestations in human envenoming. In contrast, low-abundance non-enzymatic proteins families, often classified as secondary, minor or rare components in transcriptomic and proteomic surveys, remain comparatively underexplored. Rather than acting as primary lethal effectors, these accessory proteins families are thought to modulate vascular permeability, inflammation, coagulation, pain perception or toxin spreading, and to fine-tune the activity of major toxin families [11]. Despite their modest expression levels, these accessory low-abundance proteins families can substantially increase the functional and evolutionary complexity of snake venoms and may represent important, yet underappreciated, sources of biomedical innovation.

Over the last decade, Viperidae and Elapidae (including *Bothrops*, *Crotalus* and *Micrurus* species) have been the focus of numerous omics-based studies, which have generated new information not only on venom content but also on VG biology and its biotechnological potential [9,12,13,14,15].

In this context, we performed an in-depth, multi-assembler analysis of the venom-gland transcriptomes of three medically important Colombian snakes, *Bothrops asper*, *Crotalus durissus cumanensis* and *Micrurus mipartitus*. Using four independent de novo assemblers and ToxCodAn annotations [16], followed by extensive manual curation according to their relative abundance as dominant, secondary, minor and rare, we reconstructed a high-confidence catalogue of low-abundance transcripts encoding venom proteins families (enzymatic and non-enzymatic putative proteins), while explicitly excluding glandular housekeeping transcripts. By integrating transcript abundance and gene-family composition across these species, we explore comparative patterns of proteins presence and absence as inferred from proteins encoding transcripts, thereby expanding the current view of the “hidden” venom landscape beyond the canonical dominant proteins.

## 2. Results

### 2.1. Transcriptomic Results

#### 2.1.1. De Novo Transcriptome Assembly and Quality

Venom gland transcriptomes from *Bothrops asper* (*B. asper*), *Crotalus durissus cumanensis* (*C. d. cumanensis*) and *Micrurus mipartitus* (*M. mipartitus*) from Colombia were constructed from the arrangement of four independent assemblies using Trinity v2.13.2 Kmer = 25, SPAdes v3.14.1 Kmer = 21 and SOAPTRANS v1.03 with Kmer = 31 and 97 (referred here as SDT31 and SDT97). The de novo assembly’s quality statistics, scaffold counts and N50 are shown in Table 1.

**Figure 1:**
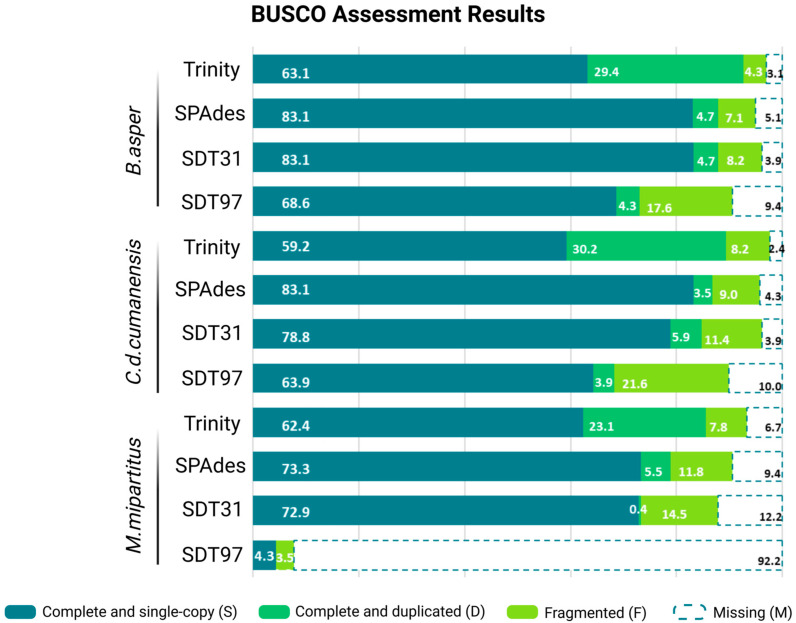
Quality assessment of each de novo assembly from the gland transcriptomes: (A) Percentage of complete and single-copy (S), complete and duplicated (D), fragmented (F), and missing (M) BUSCOs found by assembler and organism. The figure was prepared for publication using BioRender.com.

The completeness of each assembly was assessed by the Benchmarking Universal Singly Copy Orthologs (BUSCO V5.8.0.) with the eukaryota_odb10 lineage; the percentage of complete and single-copy, complete and duplicated, fragmented and missing BUSCOs from a total of 255 ortholog genes is shown in Figure 1. Trinity assembler consistently showed higher completeness (single and duplicated BUSCOs), recovering 92.5%, 89.4% and 85.5% of complete BUSCOs for *B. asper*, *C. d. cumanensis* and *M. mipartitus*, respectively. This high completeness is partly driven by a large, duplicated fraction (D = 29.4–30.2%, and 23.1% in *M. mipartitus*), consistent with a more redundant transcript set (isoforms/allelic variants/paralogs retained). SPAdes assembler achieved high completeness with much lower duplication, thus obtaining a less redundant and therefore compact assembly. SDT31 showed systematically lower structural continuity and more fragmentation/missing BUSCOs. SDT97 underperforms noticeably with much higher fragmentation and missing BUSCOs and fails to reconstruct *M. mipartitus* transcriptome by itself.

Across assemblies, BUSCO completeness and contiguity (N50) showed a consistent positive trend within each species: assemblies with higher percentages of complete BUSCOs generally exhibited higher N50 values, indicating that biologically more complete reconstructions tended to be structurally more contiguous. This pattern was most consistent for Trinity, SPAdes and SDT31, whereas SDT97 behaved as an outlier and reduced the apparent BUSCO–N50 concordance.

#### 2.1.2. Complete Protein Annotation and Curation and Protein Family Expression

**Figure 2:**
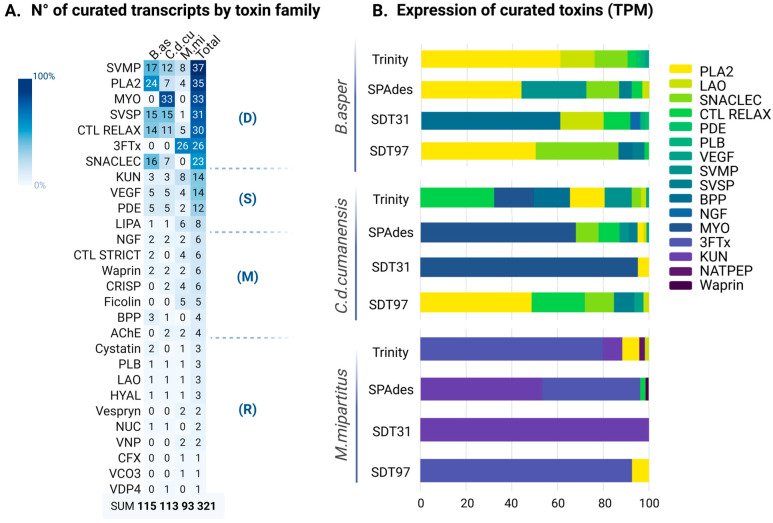
Functional annotated protein recovered from ToxCodAn and manual curation. (**A**) Curated protein transcripts count annotated in the final dataset of assemblies for each snake species (Abbreviations used in panel A: B.as = *B. asper*; C.d.cu = *C. d. cumanensis*; M.mi = *M. mipartitus*) by family and family abundance classification Dominant (D), secondary (S), minor (M) and rare (R). (**B**) Expression of the families annotated by assembly and snake species. Expression > 1% from total TPM count is shown. The figure was prepared for publication using BioRender.com.

Functional annotation of protein transcripts was performed using ToxCodAn v1.0 [16]; available at https://github.com/pedronachtigall/ToxCodAn (accessed on 21 February 2023); then, a redundancy filter was made for the final dataset. From the combined transcriptome assemblies, a total of 27 different families were identified, including dominant (high transcript count families/37-23), secondary (intermediate transcript count families/16-8), minor (low transcript count families/6-4) and rare proteins (very low transcript count families/3-2). We defined these categories solely by the number of curated protein transcripts recovered per family, and therefore they do not reflect expression levels (e.g., TPM) or imply biological relevance within the venom. Counts of unique transcripts per family recovered by assembler are shown in Figure 2A. These transcripts have complete coding sequences (CDS) and predicted signal peptides through both signal predictors SignalP v6.0 [17] and Phobius v1.05 [18]; however, transcripts that showed reduced prediction score but exhibited strong sequence homology and conserved domain architecture were retained for comparative analysis. The relevant functional domains and the characteristic number of cysteine residues for each family were also analyzed and counted. A total of 115 unique curated protein transcripts were found in *B. asper*, 113 in *C. d. cumanensis* and 95 in *M. mipartitus*. Transcript abundance is shown in Figure 2B as a percentage of the total expression of transcripts from curated protein, estimated as the read count for all transcripts as transcripts per million (TPM).

Exclusive protein transcripts were defined as those recovered by only one assembler after merging the four assemblies and collapsing redundancy by clustering. In both viperids, most exclusive transcripts were recovered by SPAdes (45.2% in *B. asper* and 46.9% in *C. d. cumanensis*), followed by Trinity (20.9% in *B. asper* and 18.6% in *C. d. cumanensis*). In contrast, for the elapid *M. mipartitus*, Trinity recovered the largest fraction of exclusive transcripts (43.8%), whereas SPAdes contributed fewer (14.3%). Across all three species, SDT31 and SDT97 contributed the fewest exclusive transcripts in all cases (2.6% and 4.3% in *B. asper*, <1% in *C. d. cumanensis* and 2.0% and 7.1% *M. mipartitus*).

Gene-expression estimated at the protein family level were strongly dependent on the assembler used (Figure 2B) and should therefore be interpreted primarily as assembly-specific reconstructions of the venom gland transcriptome rather than definitive measures of biological dominance. In *B. asper*, Trinity recovered a relatively diverse expression spectrum dominated by PLA_2_ (60.0% of total toxin TPM), LAO (14.8%) and SNACLEC (14.3%), with CTL-RELAX (3.5%) and other families each contributing <3%. In contrast, SPAdes produced a more skewed profile, with SVMP (33.5%), PLA_2_ (31.1%) and SNACLEC (17.1%) as the main components. The SDT31 assembly was strongly dominated by BPP (60.7%), followed by LAO (18.8%) and CTL-RELAX, defined in our study as CTL without canonical domains (11.6%). SDT97 yielded an intermediate-but-biased profile dominated by PLA_2_ (49.9%) and SNACLEC (36.2%), with smaller contributions from BPP (6.3%) and SVSP (5.1%), and a near absence of several families detected by the other assemblers (e.g., LAO = 0% in SDT97). These assembler-specific reconstructions nevertheless recover the same major toxin families as reported for *B. asper* venoms from Costa Rica by quantitative venomics in both Pacific and Caribbean coast specimens, respectively: SVMPs (≈44% and 41%), PLA_2_s (≈45.5% and 28.8%) and SVSPs (≈4.4% and 18.2%), and venom-gland transcriptomic surveys SVMP (≈53%), SVSP (≈20%) and PLA_2_ (≈16%) likewise report these families-together with lectin-type components and LAO within the expressed repertoire [19,20]. Importantly, these venomic percentages quantify secreted venom proteins, whereas our values represent toxin-family transcript TPM within de novo assemblies and are therefore not directly comparable beyond coarse, family-level concordance (i.e., recovery of the same major families and broadly similar dominance patterns).

In *C. d. cumanensis*, inferred dominance patterns differed markedly across assemblers: MYO accounted for most toxin TPM in SPAdes and SDT31 (67.2% and 94.4%, respectively), whereas Trinity recovered a more mixed profile where CTL-RELAX (31.8%), MYO (17.2%), BPP (15.7%), PLA_2_ (15.0%) and SVMP (11.7%) accounted for most expression. SDT97 contrasted sharply with the other assemblies, showing no MYO signal (0%) and instead being dominated by PLA_2_ (48.3%) and CTL-RELAX (23.3%), with SNACLEC (12.6%) and SVSP (8.8%) as additional major components; VEGF (3.9%) and LAO (2.5%) comprised most of the remaining expression. For this species, a proteomic profile of Colombian *C. d. cumanensis* venoms reports strong dominance of a PLA_2_ (reaching ~65% of total venom proteins), with disintegrins as a secondary fraction (~13.7%) [21]. Transcriptomic surveys across *C. durissus* subspecies likewise support a markedly PLA_2_-skewed abundance (with PLA_2_/crotoxin-related transcripts dominating toxin-coding reads), while still recovering low-abundance families such as VEGF/NGF [22].

In *M. mipartitus*, Trinity and SPAdes consistently recovered 3FTx as the dominant family (66.9–73.4%), which is consistent with proteomic analysis of *M. mipartitus* from Colombia (~60%) [23] and Ecuador (~63%) [24], with KUN (6.3–18.1%) and CTL-STRICT defined in our study as CTL with canonical domains. No other transcriptome analysis beside ours has been published on Colombian *M. mipartitus*. While venom proteomes (secreted proteins) and venom-gland transcriptomes (expressed coding repertoire) are not strictly quantitatively comparable, this agreement supports using elapid transcriptomics to contextualize venom composition and diversification [24]. Accordingly, we restrict comparisons to broad family-level trends, since transcriptomics captures gland mRNA at a sampling time point and is sensitive to assembly/mapping, whereas venomics quantifies secreted/processed proteins and is shaped by MS detectability and quantification strategy [25,26,27].

SDT31 recovered a markedly more extreme profile dominated by 3FTx (91.4%) with a smaller PLA_2_ (7.4%) fraction. SDT97 was similarly dominated by 3FTx (91.9%) and PLA_2_ (7.4%) but failed to recover the substantial KUN contribution observed in Trinity/SPAdes. Given its poorer assembly metrics in this species (low N50 and BUSCO completeness; Figure 1), the SDT97 profile for *M. mipartitus* likely reflects under-assembly and loss of transcript diversity and should therefore be considered only weakly representative of toxin-family expression estimates for the venom gland-transcriptome.

Noteworthy, families classified as dominant based on transcript counts (e.g., PLA_2_, SVMP, MYO, 3FTx, SNACLEC) generally exhibited high expression in their respective species; however, several families classified as rare according to copy number (e.g., LAO, BPP, PLB) contributed disproportionately to the expressed venom profile. LAO, for instance, is represented by only four transcripts in total (minor) but reaches 14.8–18.8% of total toxin expression in *B. asper* (yet ~1–2% in the other two species), while BPP (four transcripts) accounts for 60.7% of toxin expression in *B. asper* (SDT31 assembler) and 15.7% in *C. d. cumanensis* (Trinity assembler). Conversely, some secondary families with more copies, such as PDE, remain consistently lowly expressed (<3% across species and assemblers). These results suggest that both major and low-abundance protein families may contribute to the expressed venom gland transcriptome, although their translation, secretion and impact on the delivered venom phenotype require proteomic validation, and that transcript copy numbers alone is a poor predictor of functional dominance.

Detailed, family-specific expression patterns on non-dominant proteins and their biological implications are described in the corresponding family sections below.

### 2.2. Sequence Alignment Analysis of Non-Dominant Components by Family

#### 2.2.1. Secondary Families

##### Kunitz-Type Serine Protease Inhibitor (KSPI)

**Figure 3:**
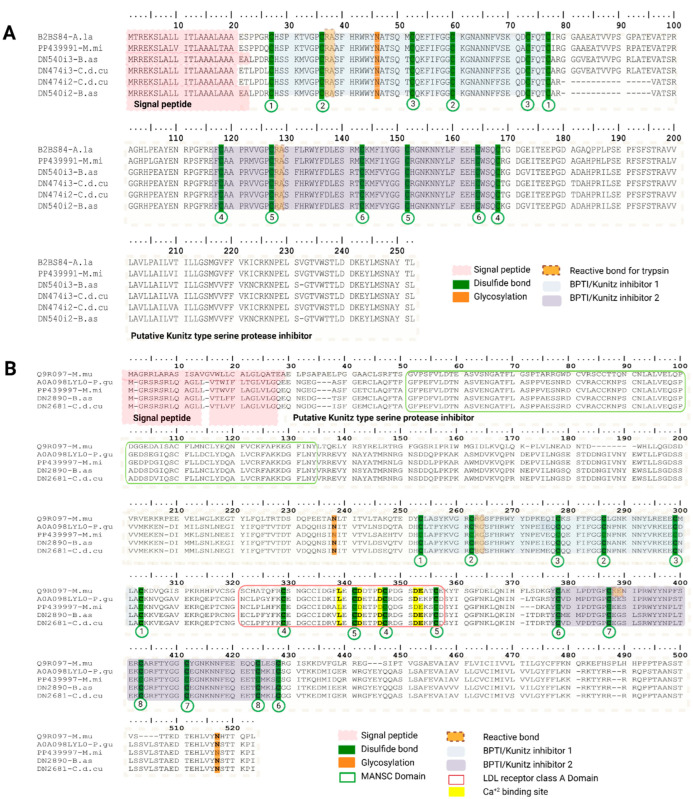
Multiple sequence alignment of: (**A**) Long putative KSPI-like transcripts from *B. asper*-B.as DN540i3-B.as (PX911394); DN540i2-B.as (PX911397), *C. d. cumanensis* DN474i3-C.d.cu (PX911395); DN474i2-C.d.cu (PX911396) and *M. mipartitus* (PP439991), with homologous sequences from *Austrelaps labialis* (B2BS84). (**B**) Multidomain putative KSPI-like transcripts from *B. asper* DN2890-B.as (PX911398) and *C. d. cumanensis* DN2681-C.d.cu (PX911399) with homologous sequences from *Pantherophis guttatus* (A0A098LYL0) and similar sequences from *Mus musculus* (Q9R097) including signal peptide, disulfide bonds, and predicted N-glycosylation sites. The figure was prepared for publication using BioRender.com.

In this study we identified a total of six KSPI-like transcripts that were grouped in two types of transcript variant: long KSPI-like (DN540i2–B.as and DN540i3-B.as from *B. asper* and DN474i2-C.d.cu and DN474i3-C.d.cu from *C. d. cumanensis*) and multidomain KSPI-like transcripts (DN2890i2-B.as and DN2681i1-C.d.cu). Eight KSPI-like transcripts from *M. mipartitus* were reviewed extensively by Saldarriaga-Cordoba et al. (2024) [28]. Long Kunitz precursors were recovered in both *B. asper* and *C. d. cumanensis* (Figure 3A). The highest hit was with a putative 252 amino acid (aa) long KSPI reviewed UniProtKB sequence from *Austrelaps labialis* (PPI of 85.0% and 81.05% for *B. asper*; PPI of 82.1% and 86.5% for *C. d. cumanensis*). Both signal peptide predictors retain the MRREKS 20 aa long in *M. mipartitus* and *C. d. cumanensis* and 22 aa long in *B. asper*. These transcripts present a mature chain with two Kunitz domains (KSPI-1 between aa ~27 to 77 and aa ~118 to 168 for KSPI-2), each with three disulfide bonds, separated by a short hydrophobic spacer described previously in *M. mipartitus* [28]. One sequence from each viperid showed 16 aa missing (DN474i2-C.d.cu and DN540i2-B.as) in this area, determining mature chains of 215 aa and 216 aa respectively, rather than the 232 aa long reviewed sequence. Function for the long Kunitz protein remains unknown experimentally.

In both *B. asper* and *C. d. cumanensis* we recovered long multi domain, type-I membrane Kunitz proteins DN2890-B.as and DN2681-C.d.cu (Figure 3B) that align to vertebrate SPINT1 (HAI-1-Q9R097; PPI of 52.1% for *B. asper* and 51.5% for *C. d. cumanensis*) and to colubrid SPINT1-like sequences (A0A098LYL0; PPI of 99.6% for *C. d. cumanensis* and 87.7% for *B. asper*). Both signal peptide predictors identified an N-terminal sequence of 25 aa long. These transcripts show the expected extracellular modular architecture (MANEC-PKD-like-Kunitz1-LDLRA-Kunitz2) followed by a single-pass transmembrane segment and short cytosolic tail. This mirrors the *M. mipartitus* SPINT1-like architecture we described previously and suggests a conserved, likely regulatory role (e.g., local control of proteolysis within the venom gland and/or prey hemostasis targets). In terms of expression, KSPI-like protein family remained consistently low in both viperids, staying below ~1% of total protein expression across all assemblies. While in *M. mipartitus*, expression of the overall family was higher, showing 6.4% in Trinity and 12% in SPAdes. In *M. mipartitus* expression was dominated by a Short KSPI-like family (98%), and the rest was split by Ku-WAP fusin-like transcripts (0.65%) and long KSPI-like transcripts (0.4%) contributing minor fractions [28]; neither short KSPI-like nor Ku-WAP fusin were detected in the viperids *B. asper* and *C. d. cumanensis*. In *M. mipartitus* multidomain KSPI-like transcripts were expressed around ~0% as well as those found in *B. asper* and *C. d. cumanensis*. Among viperids, Kunitz can be extremely scarce at the transcript level (reported only as a singleton/under-represented protein transcript in *Echis* venom gland transcriptomic data [29] and a proteomic analysis from *Bitis arietans* identifies Kunitz-type peptides among the least abundant venom protein families (<1%) [30].

##### Vascular Endothelial Growth Factor (VEGF)

VEGFs are paracrine molecules that play a physiological role widely conserved in Metazoans, which are necessary for the formation of new vessels. Angiogenesis is a fundamental process for embryonic development, somatic growth, tissue and organ healing, among others [31]. In vertebrates, VEGFs comprise VEGF-A, VEGF-B, VEGF-C and VEGF-D (also called placental growth factor or PGF). In snake venoms, a lineage-specific paralog termed VEGF-F (also svVEGF) occurs at low but variable abundance in both Viperidae and Elapidae [32], and canonical VEGF transcripts (e.g., VEGF-A or VEGF-C) can also be detected in venom-gland transcriptomes of both families [31,32].

**Figure 4:**
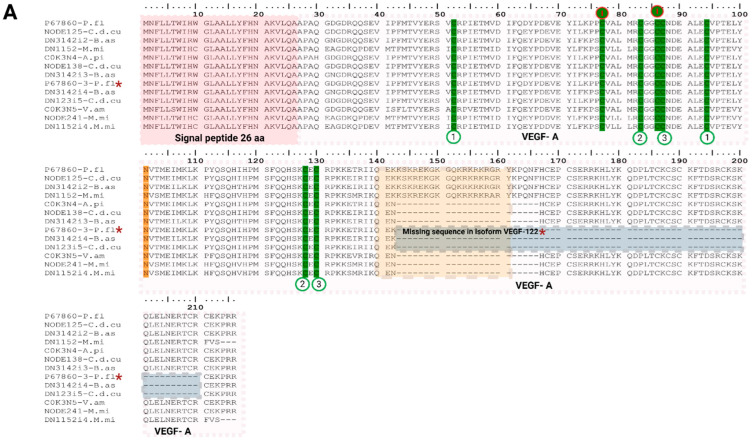

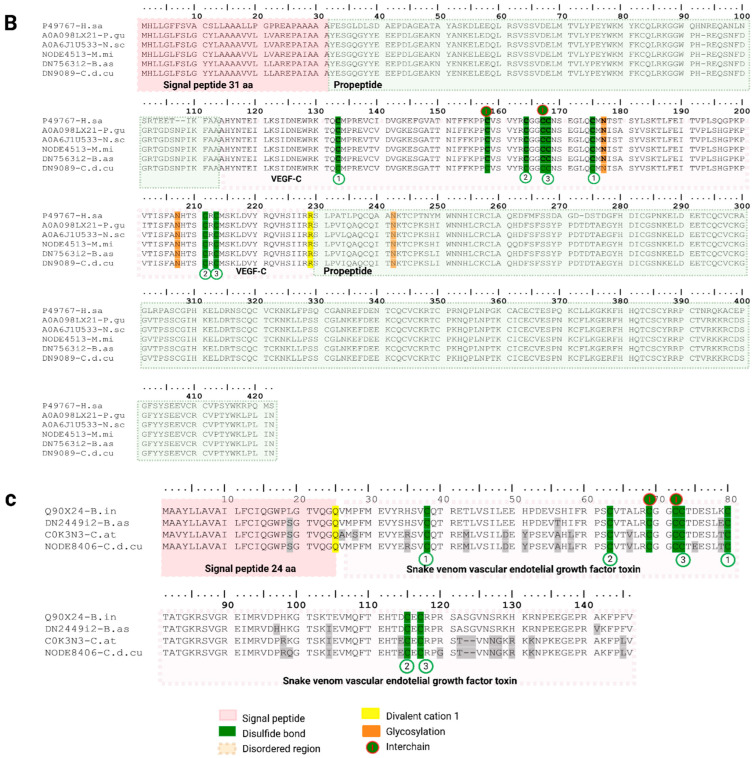
Multiple sequence alignment of: (**A**) VEGF-A-like transcripts from *C. d. cumanensis* NODE125-C.d.cu (PX911400), NODE138-C.d.cu (PX911403) and DN123i5-C.d.cu (PX911406); *B. asper* DN3142i2-B.as (PX911401), DN3142i3-B.as (PX911404) and DN3142i4-B.as (PX911405); *M. mipartitus* DN1152-M.mi (PX911402), NODE241-M.mi (PX911407) and DN1152i4-M.mi (PX911408) with homologous sequences from *Protobothrops flavoviridis* (P67860)*, Agkistrodon piscivorus piscivorus* (C0K3N4) and *Vipera ammodytes ammodytes* (C0K3N5). * *P. flavoviridis* isoform VEGF-122, lacking the sequence corresponding to residues 143-210 (**B**) VEGF-C-like transcripts from *M. mipartitus* NODE4513-M.mi (PX911409); *B. asper* DN7563i2-B.as (PX911410); *C. d. cumanensis* DN9089i2-C.d.cu (PX911411) with similar sequence from *homo sapiens* (P49767), and homologous sequences from *Pantherophis guttatus* (A0A098LX21) and *Notechis scutatus* (A0A6J1U533). (**C**) VEGF-F-like transcripts from *B. asper* DN2449i2-B.as (PX911412) and *C. d. cumanensis* NODE8406-C.d.cu (PX911413), with homologous sequences from *Bothrops insularis* (Q90X24) and *Crotalus atrox* (C0K3N3). Signal peptides, disulfide bonds, predicted N-glycosylation and a putative cation binding site are indicated. The figure was prepared for publication using BioRender.com.

In our transcriptome analysis we found three different groups of VEGFs transcript variants: VEGF-A-like, VEGF-C-like and VEGF-F transcripts. From the first group we identify a total of nine VEGF-A-like transcripts (Figure 4A), three in *B. asper* (DN3142i2-B.as, DN3142i3-B.as and DN3142i4-B.as), three in *C. d. cumanensis* (NODE125-C.d.cu, NODE138-C.d.cu and DN123i5-C.d.cu) and three in *M. mipartitus* (DN1152-M.mi, NODE241-M.mi and DN1152i4-M.mi). Across our VEGF-A-like transcripts, we observe a 26 aa signal peptide beginning with the conserved MNFLLLTWI amino acid sequence, eight Cys residues are present, of which six form three disulfide bonds at positions Cys52-Cys94, Cys83-Cys128 and Cys87-Cys130, while the remaining two form an intermolecular disulfide bridge between the homodimers at putative positions Cys77–Cys86. Three transcript patterns are evident in the alignment: (1) A full-length chain containing two domains: a platelet derived growth factor and a VEGF heparin-binding domain (HBD) with a positively charged disordered region between position 140–160, whose top BLAST v2.17.0 hit was the reviewed 216 aa (amino acid) long *Protobothrops flavoviridis* VEGF-A (P67860; pairwise percent identity (PPI) of 87.1% for *M. mipartitus*, 96.3% for *B. asper* and 99.07% for *C. d. cumanensis*), (2) the ΔHBD variants carrying a deletion within the HBD region (residues 143–166 in our alignment), with top hits to *Agkistrodon piscivorus piscivorus* (C0K3N4; PPI of 98.9% for *C. d. cumanensis* and 95.8% for *B. asper*) and *Vipera ammodytes ammodytes* (C0K3N5; PPI of 87.1% for *M. mipartitus*) and (3) isoforms homologous to TfVEGF122 identified in *Protobothrops flavoviridis* by Takahashi et al. (2004) [33], which lack the sequence corresponding to residues 143 to 210 in our alignment (P67860-3; PPI of 94.6% for *B. asper* and 98.7% for *C. d. cumanensis*). Similar ΔHBD isoforms have been reported in *Crotalus durissus terrificus*, where the deletion correlates with loss of heparin/NRP-1–dependent functions [31].

In the second group we identified three VEGF-C-like transcripts (NODE4513-M.mi, DN7563i2-B.as, DN2449i3-B.as and DN9089-C.d.cu; Figure 4B), encoding N- and C-terminal propeptides that flank the VEGF homology domain (VHD). Stepwise proteolytic processing removes both propeptides to generate the mature homodimer, which binds VEGFR-3 with high affinity (driving lymphangiogenesis) and, once fully processed, can also activate VEGFR-2 and increase vascular permeability [34]. All VEGF-C-like transcripts detected share a 31 aa signal peptide beginning with the same five amino acids MHLLG, and a cysteine knot formed by three disulfide bonds between Cys133-Cys175, Cys164-Cys211 and Cys168-Cys213. Putative N terminal (positions 32 to 113) and C-terminal (positions 230 to 422) pro-peptides are present. Transcripts also have three putative N-glycosylation sites at Asn177, 207 and 242, the first two within VHD, and one within the C-terminal pro peptide. The major C-terminal cleavage is consistent with the experimentally validated site near R227/S228 in vertebrate VEGF-C [34]. These transcripts also have high sequence identity with unreviewed sequences of snakes *Pantherophis guttatus* (A0A098LX21; PPI of ~98% for *C. d. cumanensis* and *B. asper*), and *Notechis scutatus* (A0A6J1U533; PPI of 99% for *M. mipartitus*). These three transcripts show a PPI of ~75% with the reviewed UniProtKB VEGFC from *Homo sapiens* (P49767). When we analyzed the signal peptide prediction, the SignalP v6.0 prediction probability was below the desired threshold (*p* ≥ 0.90). When we used Phobius, v1.05 the software predicted a signal peptide of 31 aa, showing consistency with the curated UniProtKB P49767 entry from *H. sapiens* which reports a 31 aa signal peptide supported by manual assertion based on experimental evidence.

The third group showed the highest hit with VEGF-F (Figure 4C); they are also homodimeric heparin-binding proteins that have a VEGF homology domain or VHD that presents between 29 to 64% of identity among the family. They maintain the eight cysteine conserved residues with a shorter and variable C-terminal region and can be classified in three types according to their interactions with VEGF receptors, heparin and NP-1 (coreceptor). In our analysis, two transcripts from *B. asper* (DN2449i2, DN2449i3-B.as) and one from *C. d. cumanensis* (NODE8406) share high identity with *Bothrops insularis* (Q90X24; PPI of 95.2%) and *Crotalus atrox* svVEGF’s (C0K3N3; PPI of 95.8%) respectively. No transcript matching VEGF family was found in *M. mipartitus*. Both VEGF-F like transcripts possess a 24 aa signal peptide, three disulfide bonds between Cys38-Cys80, Cys69-Cys115 and Cys73-127, with the homodimer linkage bond between Cys63–Cys72. The putative proteins are 122 amino acid residues long and present a higher affinity for VEGFR-1.

Regarding transcript expression of VEGF-like transcripts in the overall protein family expression, their abundance was low in the viperids species, *B. asper* (1.7%) and *C. d. cumanensis* (1.3%), while it was even lower in *M. mipartitus* (<1%). Comparing expression of VEGFs isoforms, we found that our viperid VEGF-F-like transcripts datasets account for the vast majority of VEGF-family expression, representing a 96.9% (*C. d. cumanensis*) and 94.5% (*B. asper*) of total VEGF-like family expression, whereas VEGF-A ΔHBD transcripts are minor (3.2%-*B. asper* and 2.1%-*C. d. cumanensis*) and VEGF-C/VEGF-A full-length transcripts remain <1%.

In the Elapid *M. mipartitus*, we observed VEGF-A ΔHBD transcripts as the most abundant VEGF-like isoforms (88.7% and 32.8%, respectively), with no detectable VEGF-F, whereas VEGF-C-like and full-length VEGF-A like isoforms were expressed at similar levels (11.3% and 10.3%).

##### Phosphodiesterases (svPDE)

Across Metazoa, phosphodiesterases are typically membrane-attached ectoenzymes that regulate extracellular nucleotides and thereby influence tissue damage signaling, inflammation, and platelet activation. In vertebrates, the ectonucleotide pyrophosphatase/phosphodiesterase (ENPP) family hydrolyzes ATP, ADP and/or NAD in the extracellular space, modulating purinergic signaling, coagulation, immune responses and vascular permeability [35].

**Figure 5:**
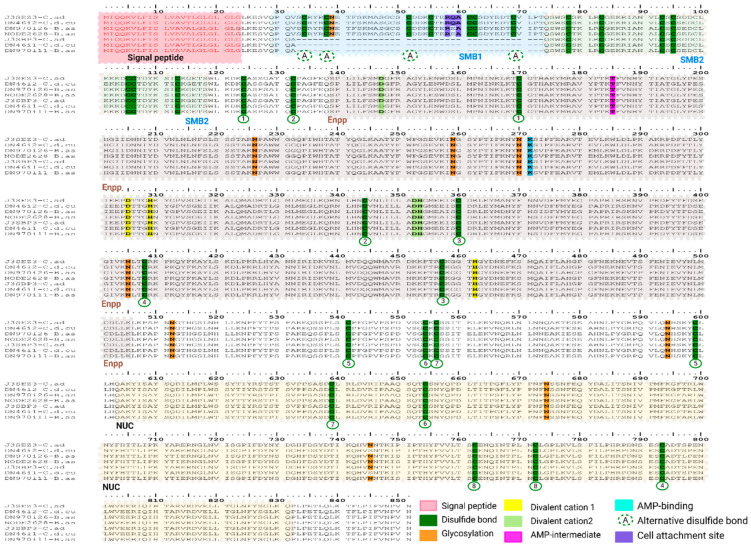
Multiple sequence alignment of PDE ENPP3-like transcripts from *C. d. cumanensis* DN46i2-C.d.cu (PX911414) and DN46i1-C.d.cu (PX911417); *B. asper* DN970i26-B.as (PX911415), NODE2628-B.as (PX911416) and DN970i11-B.as (PX911418) with homologous sequences from *Crotalus adamanteus*-(J3SEZ3-J3SBP3). Signal peptides, disulfide bonds, predicted N-glycosylation sites, among others are shown. The figure was prepared for publication using BioRender.com.

In our transcriptome analysis we identify 12 phosphodiesterase (PDE) transcripts, five in *B. asper*, five in *C. d. cumanensis* and two in *M. mipartitus*, which cluster into three ENPP-like groups. The first group includes two types of ENPP3-like transcript variants. In the first subtype, our transcripts DN46i2-C.d.cu, DN970i26-B.as and NODE2628-B.as showed hits with *C. adamanteus* svPDE1 (J3SEZ3; PPI of 99.4% for *C. d. cumanensis* and ~95.4% for *B. asper* transcripts), while in the second subtype our transcripts DN46i1-C.d.cu and DN970i11-B.as showed hits with *C. adamanteus* svPDE2 (J3SBP3; PPI of 99.4% for *C. d. cumanensis* and ~95.4% for *B. asper* transcripts); see Figure 5. They all share a 23 aa long signal peptide, consistent with the viperid ENPP3-E1a N-terminus and a mature chain of ~828 aa for the full-length form.

The modular architecture mirrors ENPP3: two Somatomedin-B domains, SMB1 (positions 30 to 73) and SMB2 (74 to 118), both cysteine-rich, disulfide-stabilized modules implicated in protein–protein interactions. Then, a PDE/ENPP catalytic domain with bi-nuclear cation^+2^ sites coordinated by Asp305, His309 and His462 for cation 1 and Asp147, Thr185, Asp352 and His353 for cation 2, plus a conserved Thr185 that coordinates the substrate (AMP). The C-terminal domain is a DNA/RNA nonspecific endonuclease (NUC) of 229 aa, shared by all the transcripts. In the svPDE2-like forms, the SMB1 domain is absent (DN46i1-C.d.cu and DN970i11-B. as) yielding a shorter mature chain (787 aa). Interestingly, we did not find an ENPP3-like transcript in *M. mipartitus* in our analysis.

**Figure 6:**
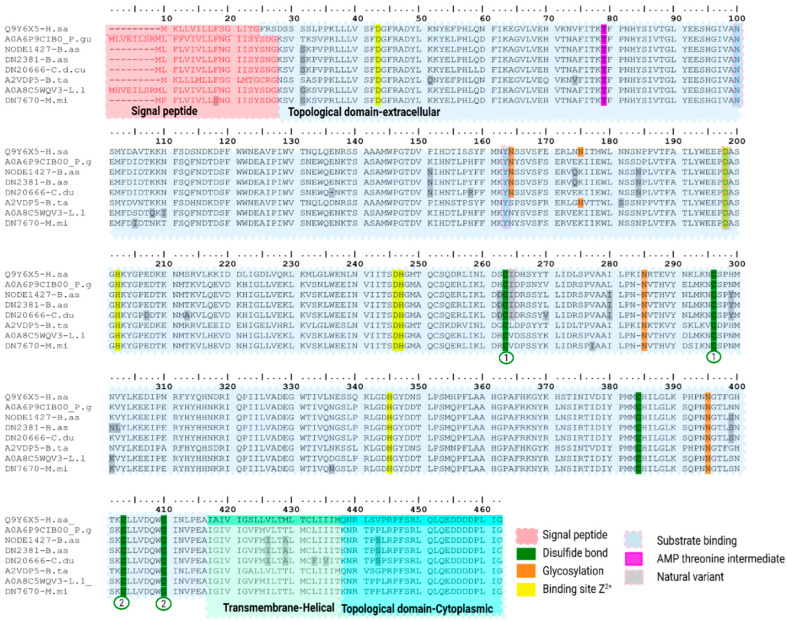
Multiple sequence alignment of PDE ENPP4-like transcripts from *B. asper* NODE1427-B.as (PX911419) and DN2381-B.as (PX911420); *C. durissus cumanensis* DN20666-C.d.cu (PX911421) with similar sequences from *Homo sapiens* (Q9Y6X5) and homologous *Pantherophis guttatus* (A0A6P9CIB0) sequences; *M. mipartitus* DN7670-M.mi (PX911422) with similar *Bos taurus* (A2VDP5) and homologous *Laticauda laticauda* (A0A8C5WQV3) sequences. Signal peptide, disulfide bonds and predicted N-glycosylation are shown, among others.

The second subtype of svPDEs identified in our transcriptome corresponds to four transcripts; three of these (NODE1427-B.as, DN2381-B.as, DN20666-C.d.cu) gave the highest hits with a Bis(5′-adenosyl)-triphosphatase ENPP4-like reviewed UniProtKB sequence from *Homo sapiens* Q9Y6X5 (PPI of ~71.0%), and unreviewed UniProtKB sequence from *Pantherophis guttatus* A0A6P9CIB0 (PPI of ~94.0%). The fourth transcript in *M. mipartitus* (DN7670-M.mi) gave the highest hit with the reviewed UniProtKB sequence A2VDP5 from *Bos taurus* (PPI of 69.6%), and the unreviewed UniProtKB sequence A0A8C5WQV3 from *Laticauda laticauda* (PPI of 97.4%). They all share a signal peptide of 19 aa and a 352 aa long catalytic core, except for the signal peptide of *M. mipartitus* transcript, which fell below the threshold in both predictors (Figure 6). This transcript was included in the alignment based on sequence homology and conservation of characteristic structural motifs. Notably, polymorphism within signal peptide regions is known to affect the in-silico prediction confidence and may account for the reduced scores observed for this transcript [17]. Human ENPP4 possesses two Zn^+2^ coordinated sites at Asp34, Thr70, Asp237 and His238, and Asp189, His193 and His336, for the second binding site shared by all transcripts and the conserved AMP-intermediate Thr79 residue. Near the C-terminal, the human ENPP4 possesses transmembrane and cytoplasmic sites that remain in our transcripts. There are currently no reviewed ENPP4-like transcript sequences from the suborder Serpentes in the UniProtKB database (accessed on 29 January 2026), and to the best of our knowledge, no ENPP4-like coding sequences have ever been documented for *B. asper*, *C. d. cumanensis* or *M. mipartitus*.

**Figure 7:**
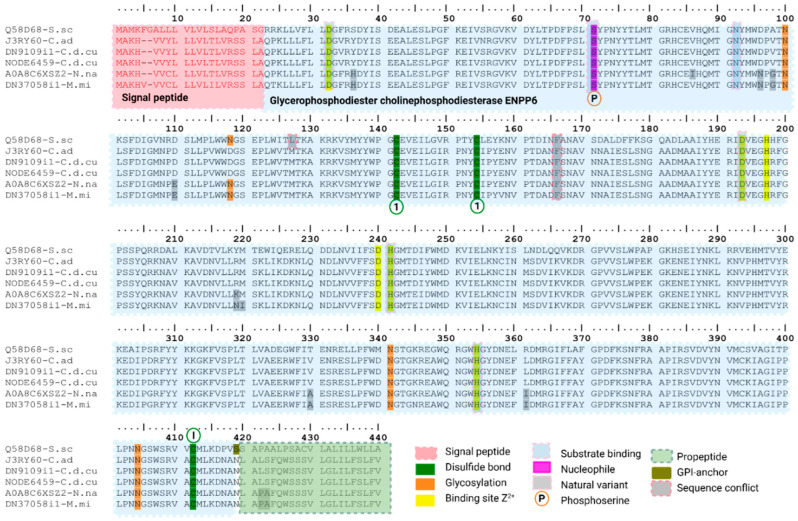
Multiple sequence alignment of PDE ENPP6-like transcripts from *C. d. cumanensis* DN9109i1-C.d.cu (PX911423) and NODE6459-C.d.cu (PX911424) with similar sequence from *Sus scrofa* (Q58D68), and homologous sequence from *Crotalus adamanteus* (J3RY60) and *M. mipartitus* DN37058i1-M.mi (PX911425) with *Naja naja* sequence (A0A8C6XSZ2). Signal peptide, disulfide bonds and predicted N-glycosylation are shown, among others. The figure was prepared for publication using BioRender.com.

In the third svPDE subtype, we identified two transcripts from *C. d. cumanensis* (DN9109i1-C.d.cu; NODE6459-C.d.cu) and one from *M. mipartitus* (DN37058i1-M.mi), matching with glycerophosphodiester choline phosphodiesterase ENPP6 identified in *Sus scrofa* (Q58D68 reviewed UniProtKB; PPI of 76%), *Crotalus adamanteus* (J3RY60 unreviewed UniProtKB sequence; PPI of 99%), for *C. d. cumanensis*, and *Naja naja* (A0A8C6XSZ2 unreviewed UniProtKB sequence; PPI of 99.7%) for *M. mipartitus* (Figure 7). They share a 20 aa residues long signal peptide, except for DN37058i1-M.mi transcript and Q58D68-S.sc that possesses 21 and 22 residues, respectively. The long mature chain for *C. d. cumanensis* is 373 aa transcript long, while the *M. mipartitus* transcript has 372 aa. They all conserve the catalytic center with two Zn^+2^ binding sites and a phospho-serine residue at position 71. In mammals, ENPP6 is a GPI-anchored ectoenzyme that hydrolyzes glycerophosphocholine (GPC) and related glycerophosphodiesters, participating in membrane-lipid recycling and showing a GPI-anchor amidated serine at position 419. Intriguingly, our snake transcripts show Asn at the position equivalent to the GPI-anchor serine. Across svPDE-related transcripts found in this analysis, ENPP3-like transcripts were the most expressed in viperids. In *B. asper* the full-length ENPP3-like isoform that accounted for 98.8% and 88.5% from the total svPDE found in their respective assemblies. However, *C. d. cumanensis* prominently expressed the 5′-truncated ENPP3-like transcript lacking the SMB1 domain, representing 64.5% of its svPDE pool, while the full-length ENPP3-like transcript contributed only 30.4%. Also, non-ENPP3 paralogs were minor in both viperids: ENPP4 and ENPP6-like expressed only 1.1% and 0.9% for ENPP4 in *B. asper* and 2.3% in *C. d. cumanensis*, and 2.7% and 2.0% for the ENPP6-like in *C. d. cumanensis* transcript. In *M. mipartitus* analysis, we do not find ENPP3-like transcripts, and expression is split between ENPP4-like (54.5%) and ENPP6-like (45.5%) transcripts (Figure 7).

##### AB Hydrolase Superfamily (LIPA)

**Figure 8:**
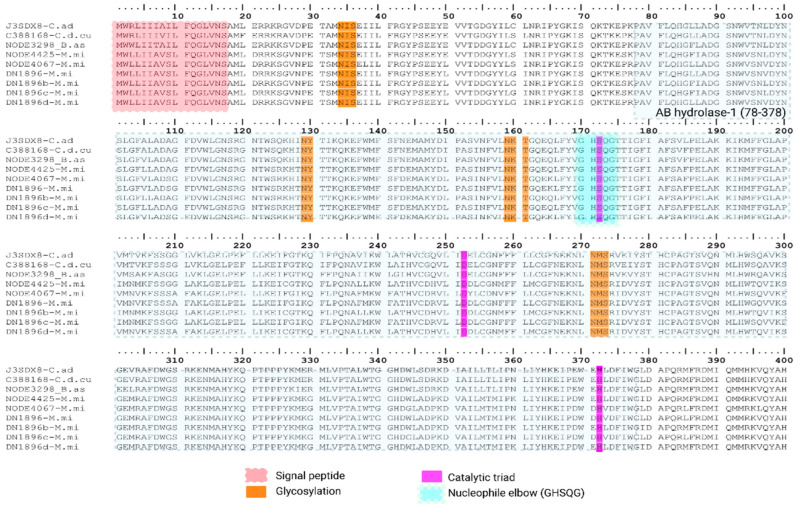
Multiple sequences alignment of LIPA from *C. d. cumanensis* C388168-C.d.cu (PX911426); *B. asper* NODE3298-B.as (PX911427); *M. mipartitus* NODE4425-M.mi (PX911428), NODE4067-M.mi (PX911429), DN1896-M.mi (PX911430), DN1896b-M.mi (PX911431), DN1896c-M.mi (PX911432) and DN1896d-M.mi (PX911433) curated lipases transcripts with homologous *Crotalus adamanteus* (J3SDX8) sequence. Signal peptides and conserved domains are indicated. The figure was prepared for publication using BioRender.com.

True triacylglycerol lipases (α/β-hydrolases) have been reported in wasp and snake venoms, but they are generally uncommon and their contribution to envenomation remains unclear [36,37]. We detected eight lipase transcripts: six in *M. mipartitus* and one each in *Bothrops asper* and *C. d. cumanensis*. All encode a 17 aa signal peptide and a 383 aa mature chain, with four conserved N-glycosylation sites (Asn34-Ile35-Ser36, Asn129-Tyr130-Thr131, Asn159-Lys160-Thr161 and Asn271-Met272-Ser273). Figure 8 shows the full alignment of hydrolase superfamily transcripts matching one hydrolase from *C. adamanteus* J3SDX8 reviewed UniProtKB sequence (PPI of 97.8% for *C. d. cumanensis*, 92.4% for *B. asper*, and ~83.5% for *M. mipartitus*). Multiple-sequence alignment against reviewed lipases places the catalytic triad at Ser172-Asp252-His372 (mature numbering: Ser155–Asp235–His355). The nucleophile lies in the canonical G-X-S-X-G (GHSQG) “nucleophile elbow” and the acidic/base residues are conserved across all copies, consistent with a bona fide α/β-hydrolase fold [38,39].

#### 2.2.2. Minor Families

##### Nerve Growth Factor (NGF)

Nerve growth factor (NGF) is a highly conserved vertebrate neurotrophin that plays central roles in the development, survival and differentiation of sympathetic and sensory neurons. These effects are mediated by activation of NGF-dependent signaling pathways, which support neuronal survival and long-range trophic communication [40,41]. Beyond the nervous system, NGF signaling is also implicated in the development and differentiation of non-neuronal tissues and in neuro-immune crosstalk [42,43]. Comparative venom-gland studies suggest that NGF has been co-opted into the advanced snake venom arsenal and subsequently retained as a typically low-copy, low-abundance component across multiple lineages, including viperids and elapids [44,45].

**Figure 9:**
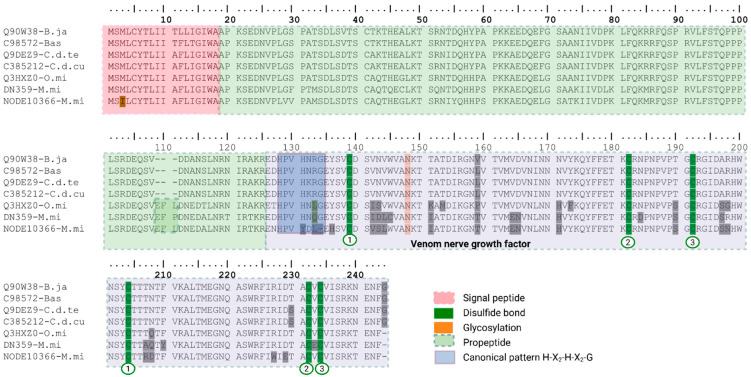
Multiple sequences alignment of NGF transcripts from *B. asper* C98572-B.as (PX911434) with homologous sequences from *Bothrops jararacussu* (Q90W38)*; C. d. cumanensis* C385212-C.d.cu (PX911435) with *C. d. terrificus* (Q9DEZ9) homologous sequence; *M. mipartitus* DN359-M.mi (PX911436) and NODE10366-M.mi (PX911437) with *Oxyuranus microlepidotus* (Q3HXZ0) homologous sequence. Signal peptides, disulfide bonds and predicted N-glycosylation sites are indicated, among others. Polimorphic sites are highlighted in gray. The figure was prepared for publication using BioRender.com.

We identified four transcripts encoding venom nerve growth factor (vNGFs) (Figure 9): one in *B. asper*, one in *C. d. cumanensis*, and two *in M. mipartitus*. The best match for the *B. asper* transcript is NGF from *Bothrops jararacussu* (Q90W38 UniProtKB reviewed; PPI of 99.2% identity), for *C. d. cumanensis* the best match is NGF from *C. durissus terrificus* (Q9DEZ9 UniProtKB reviewed; PPI of 100%), and for *M. mipartitus* the best hit is NGF from *Oxyuranus microlepidotus* (Q3HXZ0 UniProtKB reviewed; PPI of 86.8%). All transcripts contain a predicted signal peptide of 18 aa, while the mature chains consist of 223 aa in *B. asper* and *C. d. cumanensis,* and 224–225 aa in *M. mipartitus*.

Despite the presence of polymorphic sites, the conserved structural features of the mature chain, such as the N-glycosylation site and the disulfide bonds, were maintained in all transcripts when compared to the proteins from the species of their respective best hits. A notable difference was observed in the propeptide region, where the *M. mipartitus* sequences and their homolog contained three additional residues (EFL/P). Expression of this family was very low. The best expression was detected by SDTK31 with a 4.4% in *B. asper*, while the other assemblers showed an expression below 1% and Trinity did not detect expression. In both *C. d. cumanensis* and *M. mipartitus*, expression was consistently below 1%. NGF is commonly reported as a low abundance “accessory” venom component, often represented by one or few transcripts. Therefore, its relative contribution can fluctuate across assemblies and datasets (~0.5–0.7% in *Protobothrops flavoviridis* transcriptomes [46]; 0.1–1.0% in *Naja kaouthia* transcriptomes [47]).

##### Canonical/Strict C-Type Lectins (CTL)

C-type lectins belong to the broader vertebrate C-type lectin domain (CTLD) superfamily, a large group of soluble and membrane-associated proteins with diverse physiological roles, particularly in innate immunity and glycan recognition as well as cell–cell adhesion and leukocyte trafficking (like selectins) [48,49,50].

In snakes, a subset of CTLD proteins has been evolutionarily recruited into the venom system, giving rise to venom C-type lectins and related CTLD proteins [51,52]. Across advanced snakes, CTLD genes have undergone lineage-specific duplication and diversification, giving rise to (i) canonical lectins that retain key carbohydrate-recognition determinants and (ii) the more prevalent C-type lectin-like proteins (“snaclecs”), which often lose classical lectin determinants and instead mediate protein–protein interactions with hemostatic targets [51,52,53]. In this study, we operationally classify CTLD transcripts as CTL-STRICT when they conserve canonical lectin motifs, and as CTL-RELAX when those hallmark motifs are not retained.

**Figure 10:**
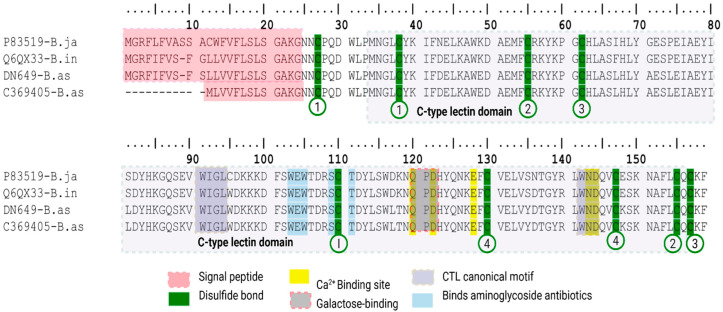
Multiple sequence alignment of two CTL-STRICT transcripts from *B. asper* DN649-B.as (PX911438) and C369405-B.as (PX911439) with homologous sequences from *B. jararacussu* (P83519) and *B. insularis* (Q6QX33). Signal peptide, disulfide bonds and canonical motifs are indicated. The figure was prepared for publication using BioRender.com.

We identified two CTL-STRICT transcripts in *B. asper* DN649-B.as and C369405-B.as (Figure 10) that showed 93% sequence identity with the C-type lectin BiL from *Bothrops insularis* (UniProtKB reviewed entry Q6QX33) and 87.4% identity with a galactose binding CTL from *B. jararacussu* (UniProtKB reviewed entry P833519) whose crystallographic structure has been experimentally validated [54]. C369405-B.as transcript encodes a shorter predicted signal peptide (13 aa) compared with DN649-B.as transcript and Q6QX33 sequence of *B. insularis* (23 aa). The predicted deletion affects both a positively charged N-terminal and hydrophobic regions of the signal peptide and could potentially affect protein targeting, translocation, processing and stability [55]; however, no functional evidence is available for this transcript. These *B. asper* transcripts are considered as CTL-STRICT because they maintained the hallmark motifs WIGL, QPD, and WND, which define classical snake venom CTLs. QPD is typically associated with galactose-type specificity, consistent with the annotation of the closest curated homologs. CTLs transcripts that showed non canonical hallmark motifs (CTL-RELAX) will be reviewed elsewhere.

**Figure 11:**
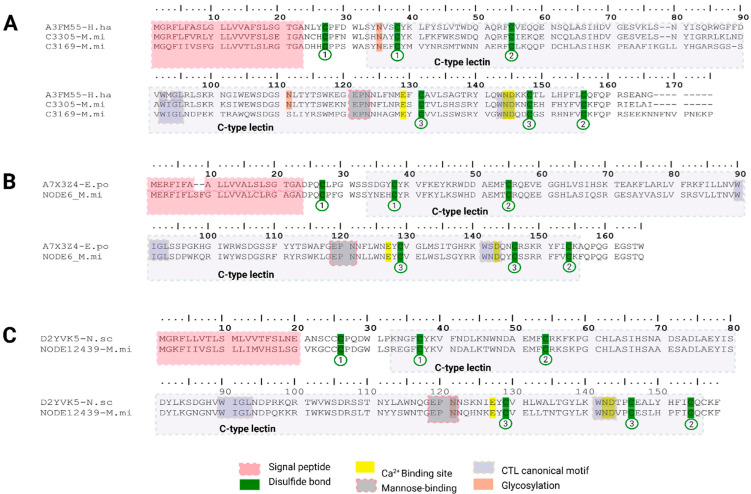
Multiple sequence alignment of four CTL-STRICT transcripts from *M. mipartitus* C3305-M.mi (PX911440), C3169-M.mi (PX911441), NODE6_M.mi (PX911442) and NODE12439-M.mi (PX911443) with homologous sequences from (**A**) *Hydrophis hardwickii* (A3FM55), (**B**) *Erythrolamprus poecilogyrus* (A7X3Z4) and (**C**) *Notechis scutatus* (D2YVK5). Conserved motifs, disulfide bonds, calcium- and aminoglycoside-binding sites, and predicted N-glycosylation sites are indicated. The figure was prepared for publication using BioRender.com.

For *M. mipartitus*, we identified four transcripts encoding CTL-STRICT characterized by the conserved motifs (corresponding to aa sequences) WIGL, EPN and WND (Figure 11). The presence of the EPN motif is consistent with mannose-binding specificity, as inferred from similarity with manually curated UniProt entries annotated by sequence homology. Two transcripts are predicted to encode 23 aa signal peptides (C3305-M.mi, C3169-M.mi) showed similarity to C-type lectin 1 from *Hydrophis hardwickii* (reviewed UniProt entry A3FM55, 68.9% identity) (Figure 11A). Another transcript predicted to encode 23 aa signal peptide (NODE6-M.mi) showed identity with lectoxin-Lio1 from *Erythrolamprus poecilogyrus* (reviewed UniProt entry A7X3Z4, 63.4% identity) (Figure 11B), and the remaining transcript predicted to encode 20 aa signal peptide (NODE12439-M.mi) matched a mannose-binding C-type lectin isoform from *Notechis scutatus scutatus* (reviewed UniProt entry D2YVK5) (Figure 11C). In terms of expression CTL-STRICT is very underrepresented in both viperid snakes (below 1%), compared to CTL-RELAX transcripts, showing expression as high as 11.6% in *B. asper* (by SDT31) and 31.8% in *C. d. cumanensis* (by Trinity). These were reversed in *M. mipartitus,* where CTL-STRICT expression peaked in SPAdes with 2.2% from the total protein expression. CTL-RELAX were all expressed below 1%.

##### Waprin

Waprins are small secreted, cysteine-rich proteins that contain at least one Whey Acidic Protein (WAP) domain (~50–60 aa) with a four-disulfide core formed by 8 conserved cysteines [56]. In Humans WFDC (WAP paralog) such as elafin, SLPI or HE4 that regulate proteases and have antimicrobial and immunomodulatory roles that protect mucous membranes [57,58].

Within snake venoms, waprins have been described mostly in Elapids and are generally low-abundance components. Functional assays showed no neuro- or hemotoxicity and clear antibacterial activity (primarily against Gram-positive bacteria) for omwaprin and nawaprim from *Oxyuranus microlepidotus* and *Naja nigricollis*, respectively [59,60].

**Figure 12:**
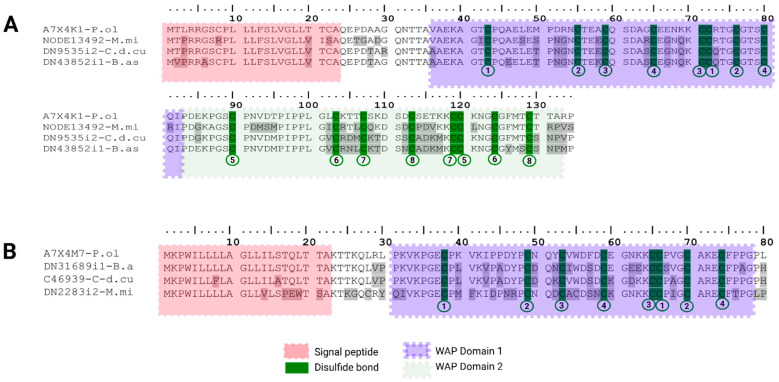
Multiple sequence alignment of Waprin transcripts from *M. mipartitus* NODE13492-M.mi (PP439998) and DN2283i2-M.mi (PP439999); *C. d. cumanensis* DN9535i2-C.d.cu (PX911445) and C46939-C-d.cu (PX911448); *B. asper* DN43852i1-B.as (PX911446) and DN31689i1-B.as (PX911447) with homologous sequences from *Philodryas olfersii* (P.ol) (**A**) Double WAP isoform (A7X4K1). (**B**) Single WAP isoform (A7X4M7). Signal peptides, disulfide bonds and conserved domains are indicated. Polimorphic sites are highlighted in gray. The figure was prepared for publication using BioRender.com.

In this transcriptome analysis, we retrieve six waprin-like transcripts, two from each snake species, segregated into two groups: Double and single WAP transcripts. Double type WAPs (DN9535i2-C.d.cu, DN43852i1-B.as and NODE13492-M.mi- accession number PP439998 published by [28]) matched with Waprin-Phi1 A7X4K1 UniProtKB reviewed from *Philodryas olfersii* (PPI of 77.6% for *C. d. cumanensis*, 75.4% for *B. asper* and 74.6% for *M. mipartitus*). These show 23 aa signal peptides and 111 aa long putative mature chains. The WAP domain 1 is at position 36 to 82 and WAP domain 2 is at position 83 to 134, each one preserving the 8-Cys core (Figure 12A).

Single WAP-like transcripts (DN31689i1-B.as, C46939-C-d.cu and DN2283i2-M.mi- accession number PP439999 published by [28]) matched with Waprin-Phi3 A7X4M7 UniProtKB reviewed from *Philodryas olfersii* (PPI of 82.3% for *C. d. cumanensis*/*M. mipartitus,* and 68.0% for *B. asper*). These sequences have a 22 aa signal peptide and a 58 aa mature chain with four putative disulfide bond cores between Cys38-Cys66, Cys49-Cys70, Cys54-Cys65 and Cys59-Cys74 (Figure 12B). In the two viperids, waprins were consistently rare or undetected. In contrast, *M. mipartitus* displayed substantially higher waprin expression, reaching 1.9% (Trinity) and 2.4% (SPAdes), consistent with the accessory/low-abundance attributes described before for this family [46,47,61].

##### Cysteine-Rich Secretory Proteins (CRISP)

Snake venom cysteine-rich secretory proteins (svCRISP) are ionic channel blockers (L-type Ca+2 and CNG). They increase vascular permeability and inhibit angiogenesis [62,63]. Structurally, they are secreted proteins with an N-terminal CAP (a.k.a., SCP) domain, a short hinge, and a C-terminal cysteine-rich region. Mature CRISPs typically contain about eight disulfide bonds that stabilize these modules [64].

**Figure 13:**
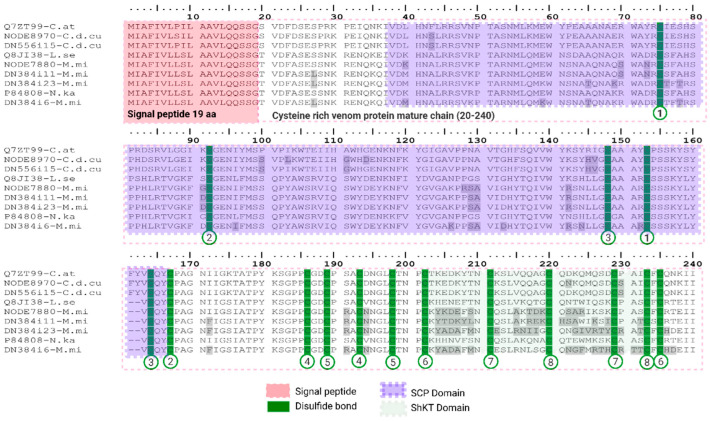
Multiple sequence alignment of CRISP from *C. d. cumanensis* NODE8970-C.d.cu (PX911450) and DN556i15-C.d.cu (PX911451) with homologous sequences from *C. atrox* (Q7ZT99); *M. mipartitus* NODE7880-M.mi (PX911452), DN384i11-M.mi (PX911453), DN384i23-M.mi (PX911454) and DN384i6-M.mi (PX911455) with homologous sequences from *Laticauda semifasciata* (Q8JI38) and *Naja kaouthia* (P84808). Signal peptides, disulfide bonds and conserved SCP and ShKT domains are indicated. Polimorphic sites are highlighted in gray. The figure was prepared for publication using BioRender.com.

We detected six CRISP-like transcripts in the venom-gland transcriptomes of *M. mipartitus* (four) and *C. d. cumanensis* (two). We recovered no CRISP-like transcript from *B. asper* in these assemblies. All carry a 19 aa signal peptide and a conserved CAP domain (Figure 13). *C. d. cumanensis* transcripts (NODE8970-C.d.cu, DN556i15-C.d.cu) present the highest identity with cysteine-rich venom protein catrin from *Crotalus atrox* (Q7ZT99 UniProtKB reviewed; PPI of 96.3% and 9.6%) and encode a ~221 aa mature chain with a putative Sperm-Coating Protein domain (SCP) or CAP domain from aa position 38 to 166 (substrate/ligand-binding cavity). The CAP domain is followed by a ShK-toxin (ShKT) domain (named after the sea-anemone peptide ShK, implicated in ion channel modulation); the motif responsible for ionic channel modulation corresponds to aa positions 202 to 235. *M. mipartitus* transcripts (NODE7880-M.mi, DN384i11-M.mi, DN384i23-M.mi) showed the highest hit with cysteine-rich venom protein latisemin from *Laticauda semifasciata* (Q8JI38 UniProtKB reviewed; PPI of 87%, 83.6% and 80.7%, respectively) showing a 219 aa mature chain. The fourth *M. mipartitus* transcript (DN384i6-M.mi) showed the highest hit with Kaouthin-2 from *Naja Kaouthia* (P84808 UniProtKB reviewed; PPI of 80.7%). In all cases the transcripts preserved the CAP and ShKT-like architecture, except for two missing amino acids in the CAP domain (Figure 13).

Expression of CRISP-like transcripts against overall protein expression in the three snake species is near 0%. Even though the CRISP family is widely distributed across front-fanged snakes, they typically present low abundance at transcriptomic and proteomic levels [47,65,66]. Comparative studies indicate no clear phylogenetic trend in CRISP abundance between viperids and elapids; rather, expression varies within species and even among populations [67].

##### Ficolin-like/”Veficolins”

To date, ficolin-like proteins in snake venoms have only been reported in *Cerberus rynchops*, where a novel family called Veficolins was identified using transcriptomic and proteomic approaches [68]. We report ficolin-like transcripts in venom gland assemblies, but their translation/secretion remains to be validated.

**Figure 14:**
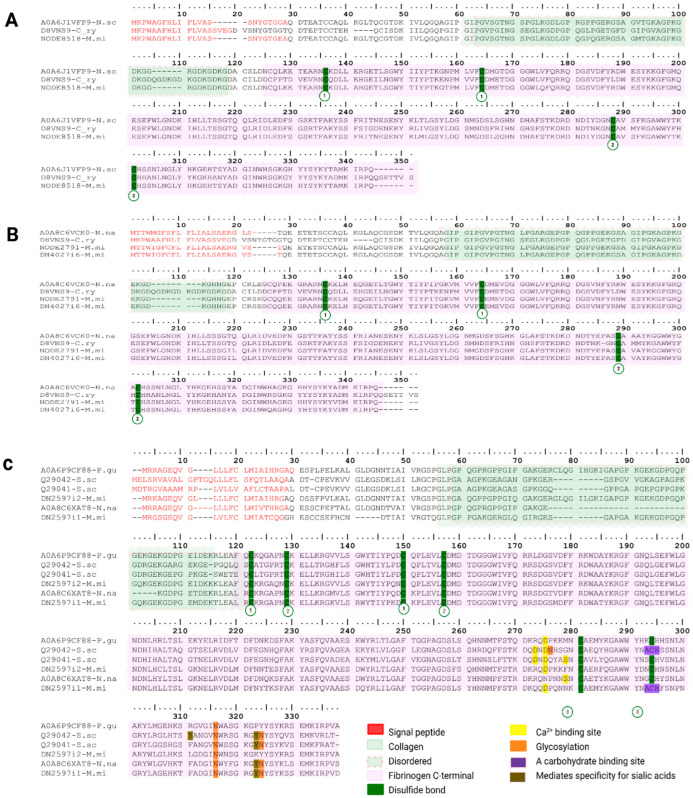
Multiple-sequence alignment of ficolin-like proteins of (**A**) *M. mipartitus* NODE8518-M.mi (PX911456) ficolin with homologous *Cerberus rynchops* (D8VNS9) and *Notechis scutatus* (A0A6J1VFP9) sequence. (**B**) *M. mipartitus* NODE2791-M.mi (PX911457) and DN4027i6-M.mi (PX911458) with homologous sequence from *Naja naja* (A0A8C6VCK0). (**C**) *M. mipartitus* DN2597i2-M.mi (PX911459) and DN2597i1-M.mi (PX911460) with homologous sequences from *Pantherophis guttatus* (A0A6P9CF88) and *Naja naja* (A0A8C6XAT8) and similar *Sus scrofa* (Q29042 and Q29041) sequences. The figure was prepared for publication using BioRender.com.

We only identified five transcripts encoding this family in the *M. mipartitus* venom gland transcriptome (NODE8518-M.mi, NODE2791-M.mi, DN4027i6-M.mi, DN2597i1-M.mi and DN2597i2-M.mi). The signal peptide length was 23 aa in all transcripts, except for DN2597i1-M.mi, which encodes a 22 aa signal peptide. The length of the mature polypeptide varies across transcripts, ranging from 296 residues in DN2597i1-M.mi to 310 residues in DN2597i2-M.mi (Figure 14A,B). Three transcripts showed the best hit to the UniProtKB reviewed sequence Ryncolin-3 from *Cerberus rynchops* (D8VNS9; PPI of 72.1% for NODE8518-M.mi transcript and 62% for NODE2791-M.mi and DN4027i6 transcripts). This reference sequence contains 19-residue signal peptides and a 328-residue mature chain. Additionally, these transcripts revealed hits to UniProtKB unreviewed sequences A0A6J1VFP9 from *Notechis scutatus* (NODE8518-M.mi, PPI of 96.7%) and A0A8C6VCK0 from *Naja naja* (NODE2791-M.mi, PPI of 95.5%; DN4027i6-M.mi, PPI of 94.3%). Alignment against a curated *Cerberus rynchops* ficolin included the expected two disulfide-bonded N-terminus, a collagen-like stalk and a C-terminal fibrinogen-like (FBG/FReD) domain.

The remaining transcripts presented the best hit to the UniProtKB reviewed sequences Q29041, Ficolin-2 (DN2597i1-M.mi, PPI of 62.1%) and Q29042, Ficolin-1 (DN2597i2-M.mi, PPI of 57.1%) from *Sus scrofa*. These reference sequences contain 26 aa and 29 signal peptides, respectively. Both have a mature chain length of 297 aa. Additionally, these transcripts showed hits to UniProtKB unreviewed sequences A0A6P9CF88, Ficolin-1-like from *Pantherophis guttatus* (DN2597i2-M.mi, PPI of 85.9%) and A0A8C6XAT8, fibrinogen C-terminal domain-containing protein from *Naja naja* (DN2597i1-M.mi, PPI of 76.1%). Alignment against a curated *Sus scrofa* ficolin also reveals the expected three disulfide-bonded N-terminus, a collagen-like stalk, a C-terminal fibrinogen-like (FBG/FReD) domain, Glycosylation sites, Ca^2+^ binding sites, a carbohydrate binding site, specificity for sialic acids and a P domain (Figure 14C). In terms of expression, ficolins were recovered only in *M. mipartitus* and remained a minor component (<1% of total protein expression), consistent with reports that ficolin-like transcripts are often lowly expressed and may fail proteomic verification even when present in venom-gland transcriptomes [68].

##### Bradykinin Potentiating Peptide Family (BPPs)

Bradykinin-potentiating peptides (BPPs) are short peptides first described in *Bothrops jararaca* venom in the 1960s by Sérgio Ferreira [69] and in scorpion venoms and amphibian skin secretions [70,71].

**Figure 15:**
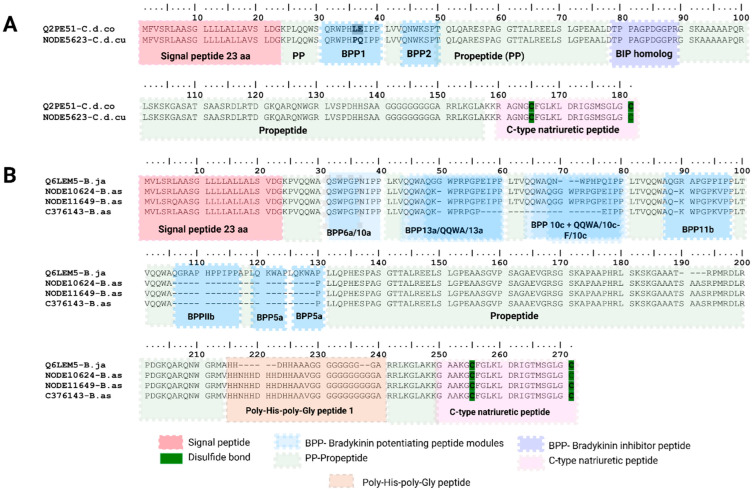
Multiple sequence alignment of BPP transcripts identified in (**A**) *C. d. cumanensis* NODE5623-C.d.cu (PX911461) with the homologous sequence from *C. durissus collineatus* (Q2PE51). (**B**) *B. asper* NODE10624-B.as (PX911462), NODE11649-B.as (PX911463) and C376143-B.as (PX911464) with homologous sequence from *B. jararaca* (Q6LEM5). The figure was prepared for publication using BioRender.com.

Our transcriptome analysis revealed multimodular BPP/CNP precursors in *C*. *d. cumanensis* (NODE5623-C.d.cu) and *B. asper* (NODE10624-B.as, NODE11649-B.as, C376143-B.as) comprising a 23 aa signal peptide followed by a mature chain in which multiple BPP modules are arranged in tandem (Figure 15). These modules are separated by propeptide spacer regions, consistent with a precursor organization that allows proteolytic processing into individual bioactive peptides [72]. A comparison of the signal peptide sequence between *C. d. cumanensis* and *B. asper* showed differences at the N- and early H-domains: MFV aa residues in *Crotalus* and MVL residues in *Bothrops*. In one *B. asper* BPP copy (NODE11649-B.as), an L→Q substitution was detected at position 6 of the 23-residue signal peptide, resulting in a decrease of the total hydrophobic ratio from 76% to 71%. Although this substitution clearly reduces the hydrophobicity of the H-domain, its impact on the secretion efficiency of the protein remains unknown [73].

The mature chain of transcripts ranged from 202 to 222 aa in *B. asper* copies and 158 aa *in C. d. cumanensis*. The BPP copy identified in *C. d. cumanensis* (NODE5623-C.d.cu) shared 98.9% identity with a homolog from *C. durissus collineatus* (Q2PE51 UniProtKB reviewed sequence) (Figure 15A). Alignment analysis revealed that the only difference was restricted to the bradykinin-potentiating peptide-1 region, where *C. d. collilineatus* contain the LE residues and *C. d. cumanensis* presents PQ residues. In addition, we identify an 11 aa Bradykinin inhibitor peptide (TPPAGPDGGPR) located between the bradykinin-potentiating peptide and C-type natriuretic peptide domains.

The three *B. asper* BPP transcripts, which encode the multimodular precursor proteins, showed the highest identity to *B. jararaca* (Q6LEM5 UniProtKB reviewed sequence), with an overall identity of 80% (Figure 15B). Sequence comparison revealed differences between BPP modules, except for BPP-6a and BPP-10a, which were conserved. One *B. asper* copy (C376143-B.as) lacked BPP13a and BPP10c, and all three lacked BPP IIb and BPP5a (NODE10624-B.as, NODE11649-B.as, C376143-B.as).

BPP transcripts are well known for being complicated to recover for de novo assembly/annotation because of proline-rich, low-complexity segments and repeated motifs. Expression of BPP/CNP family was detected by neither Trinity nor SPAdes in *B. asper* but dominated in SDT31 protein expression profile (60.7%) while in SDT97 it corresponded to only a 6.3% from total protein expression. Conversely, in *C. d. cumanensis*, BPPs were substantially found by Trinity (15.69%) but dropped to <1% with SPAdes and were not detected by SDT31/SDT97. In *M. mipartitus*, BPPs were not detected in any assembly, consistent with previous findings that it is not a protein normally detected in *Micrurus* genus [74]. Unlike viperids, in elapids NP can be found as a single transcript not attached to BPP. BPP/CNP precursors have been characterized in viperids and in the *Bothrops* genus can rank among the most highly low-abundant components expressed venom gland as reported in *B. jararaca* [75,76]. Expression in both viperids among the transcripts of the family was contributed by only one copy: the large BPP one in *B. asper* and the smaller one in *C. d. cumanensis*.

##### Acetylcholinesterase (AChE)

Acetylcholinesterase (AChE) is a member of a specific family of serine hydrolases and is distributed in both synaptic and extra-synaptic compartments. In synaptic regions, AChE is primarily responsible for the rapid breakdown of the neurotransmitter acetylcholine (ACh), whereas its physiological role outside synapses remains poorly understood [77].

**Figure 16:**
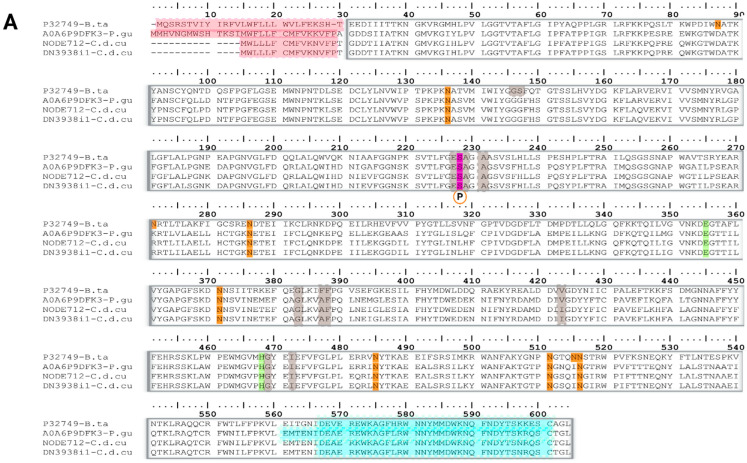

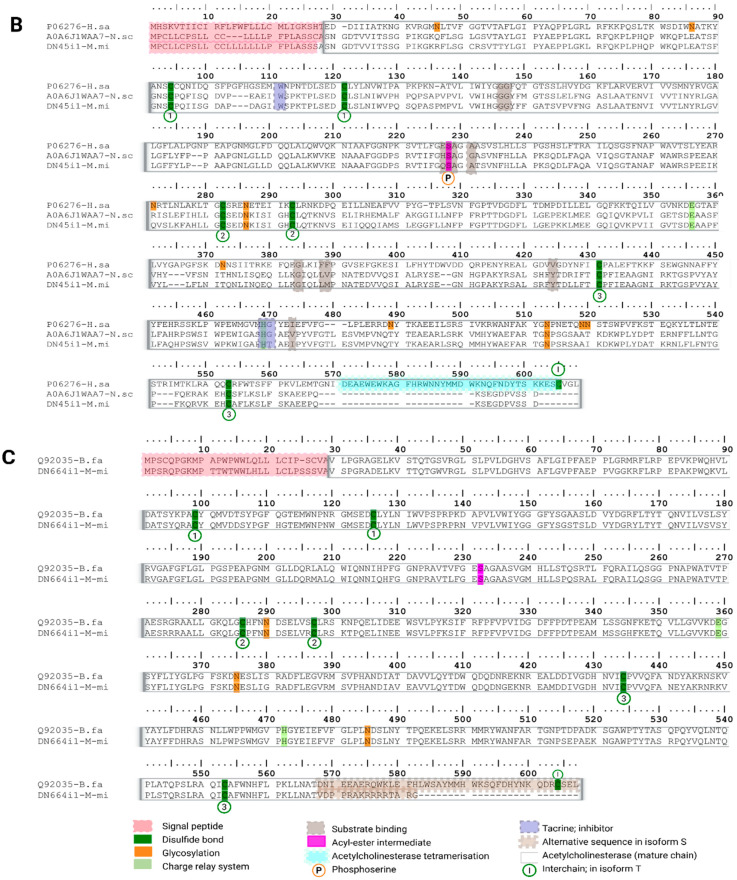
(**A**) Multiple sequence alignment of AChE transcripts from (**A**) *C. d. cumanensis* NODE712-C.d.cu (PX911465) and DN3938i1-C.d.cu (PX911466) with similar sequences from *Bos taurus* (P32749) and homologous *Pantherophis guttatus* (A0A6P9DFK3). (**B**) *M. mipartitus* DN45i1-M.mi (PX911467) with similar sequences from *Homo sapiens* (P06276) and homologous *Notechis scutatus* (A0A6J1WAA7). (**C**) *M. mipartitus* DN664i1-M.mi (PX911468) with homologous sequences from *Bungarus fasciatus* (Q92035). Signal peptides and conserved domains are indicated. The figure was prepared for publication using BioRender.com.

From the Viperidae family, only two AChE-like transcripts were detected in *C.d. cumanensis* (Figure 16A; NODE712-C.d.cu and DN3938i1-C.d.cu) that showed highest identity with cholinesterase from *Bos taurus* (P32749 UniProtKB reviewed, PPI of ~69%) and unreviewed UniProtKB sequence of *Pantherophis guttatus* carboxylic ester hydrolase A0A6P9DFK3 (PPI of ~91%). Signal peptide motifs were not conserved, as both *B. taurus* and *P. guttatus* have 30 aa signal peptides (with ~574 aa mature chains), while *C. d. cumanensis* AChE-like transcripts have 16 aa putative signal peptides and ~588 aa mature chains. The mature chain retains all functional motifs: The catalytic core is composed of a charge relay system with Ser228/E355/His468, with a putative phosphoserine modification at position 228. Main putative N-glycosylation motifs are conserved across these species, except for the Asn87, Asn271 and Asn514 positions, which are present in *B. taurus* but absent in *P. guttatus* and *C. d. cumanensis* (Figure 16A). At the C-terminal a tetramerization site from 566–601 in both *C. d. cumanensis* sequences was retained, consistent with the AChE-T isoform tetramerization segment (T-peptide), rather than venom specific AChE S-isoforms found in the elapids like *Bungarus fasciatus, B. multicintus, B. suzhenae* and *B. bungaroides*, among others, suggesting that in *C. d. cumanensis* cholinesterases are expressed in tissues rather than being part of the venom arsenal [78,79,80].

Interestingly, in *M. mipartitus* we identified two distinct types of AchE transcripts. As shown in Figure 16B, the highest hit corresponded to the UniProtKB reviewed *Homo sapiens* cholinesterase P06276 (PPI of 39.4%) and an unreviewed UniProtKB sequence carboxylic ester hydrolase from *Notechis scutatus* (PPI of 85.6%). These transcripts share a conserved AChE structural architecture like that described above for *C. d. cumanensis*, including three conserved disulfide bonds, a catalytic core comprised by a charge relay system with a putative phosphoserine modification, and two N-glycosylation sites. However, they differ at the C-terminal tetramerization domain, which is absent in both *N. scutatus* and *M. mipartitus* sequences.

Finally, as shown in Figure 16C, a soluble “true venom” AChE transcript was identified in *M. mipartitus*, showing the highest sequence similarity to *Bungarus fasciatus* acetylcholinesterase (UniProtKB reviewed Q92035; PPI of 88%). The overall sequence alignment revealed strong conservation of both catalytic and structural motifs, although the *M. mipartitus* sequence exhibits a shorter C-terminal region compared to that reported for *B. fasciatus*. This alternative sequence in isoform S “VDPPRAD(K)RRRRS(T)ARA(G)” was reported by Cousin et al. (1996) [81]. Expression of the AchE transcript family accounted for less than 1% in all assemblies.

#### 2.2.3. Rare Families

##### Cystatins

Functional cystatin inhibition relies on three structural motifs: (1) a flexible N-terminal region, with a conserved Gly-6 residue (double-G motif) that forms one of the primary contact points with the cysteine protease; (2) the central QXVXG motif (loop 1 or “central arm”), essential for binding the active-site cleft; and (3) the C-terminal PW dipeptide (Pro124-Trp125 in our alignment), forming loop 2, a second anchoring element required for high-affinity inhibition [82,83].

**Figure 17:**
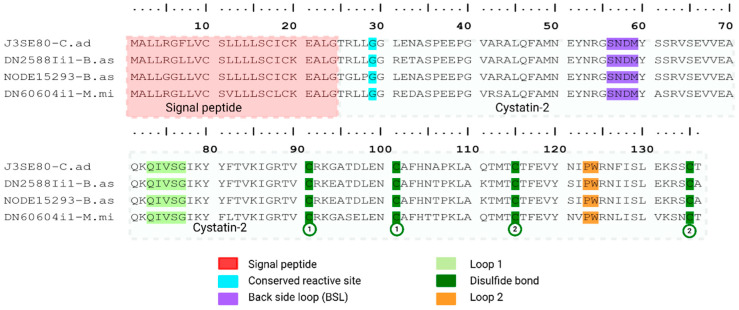
Multiple sequence alignment of cystatin-like transcripts from *B. asper* DN2588i1-B.as (PX911469) and NODE15293-B.as (PX911470); *M. mipartitus* DN60604i1-M.mi (PX911471) with homologous sequences from *Crotalus adamanteus* (J3SE80). Signal peptides, disulfide bonds and conserved motifs are indicated. The figure was prepared for publication using BioRender.com.

In our transcriptome we detected three cystatin-like transcripts. One transcript from *M. mipartitus* (DN60604il-M.mi) and two from *B. asper* (DN2588Iil-B.as, NODE15293-B.as) presenting the highest identity with cystatin-2 from *Crotalus adamanteus* (J3SE80 UniProtKB reviewed; PPI of 93.3% and 92.6% for *B. asper* transcripts; PPI of 86.8% for the *M. mipartitus* transcript). These sequences share 24 aa signal peptides, and the mature chains are 111 aa long in *B. asper* and 112 aa in *M. mipartitus*. Their architectures are consistent with family-2 cystatins, showing two intramolecular disulfide bonds (Cys91–Cys101 and Cys115–Cys135 in our alignment), as illustrated in Figure 17.

The three transcripts maintain the canonical tripartite inhibitory wedge that defines cystatin biological function. Specifically, the flexible N-terminal region, where the conserved Gly-5 residue (mature protein) essential for binding to the proteinase target conserved region (SNDM) that is implicated in legumain inhibition, named back side loop (BSL); the central QXVXG motif (QIVSG in our alignment), which forms the core of loop 1; and the C-terminal PW motif (Pro124-Trp125), responsible for loop 2 anchoring, are all preserved without substitutions. The expression of cystatin transcripts against the overall protein expression in the three snake species is near 0%, as seen in *Micrurus* venomics, where they represent ~0–0.02% of the transcriptomes [61], leading to the interpretation that their weak expression is more compatible with housekeeping/regulatory roles (protease control and maintenance of venom-gland/venom integrity) than with primary toxic function [84].

##### Phospholipase B (PLB)

Phospholipase B is a large lipolytic enzyme that hydrolyzes both acyl ester bonds of glycerophospholipids and displays lysophospholipase activity [85].

**Figure 18:**
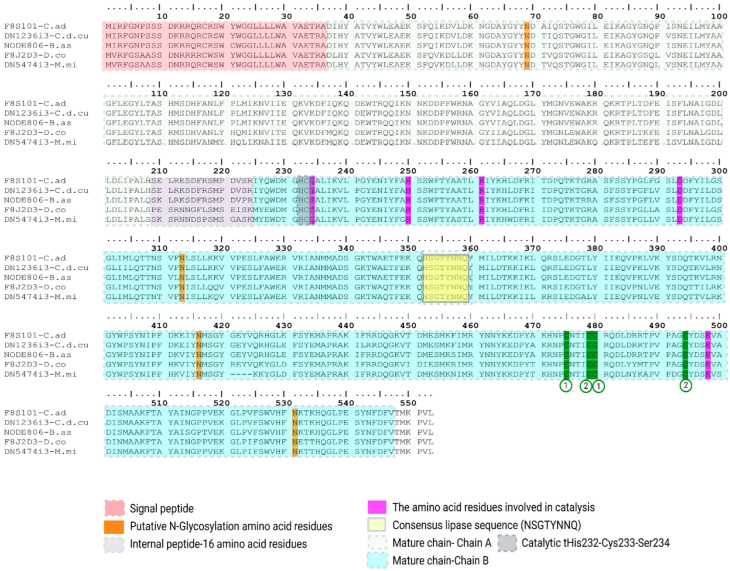
Multiple sequence alignment of PLB transcripts from *C. d. cumanensis* DN1236i3-C.d.cu (PX911472); *B. asper* NODE806-B.as (PX911473) with homologous sequences from *Crotalus adamanteus* (F8S101); *M. mipartitus* DN5474i3-M.mi (PX911474) with *Drysdalia coronoides* (F8J2D3). Signal peptides, disulfide bonds and conserved domains are indicated. The figure was prepared for publication using BioRender.com.

We detected three PLB-like transcripts in our transcriptome analysis, one transcript in each species analyzed (Figure 18). The viperid transcripts (DN1236i3-C.d.cu, NODE806-B.as) matched with Phospholipase B from *Crotalus adamanteus* (F8S101 UniProtKB reviewed, PPI of 98.2% and 96.9% respectively) and share a 36 aa signal peptide and a 517 aa mature chain. *M. mipartitus* transcript (DN5474i3-M.mi) matched with phospholipase-B 81 from *Drysdalia coronoides* (F8J2D3 UniProtKB reviewed, PPI of 91.2%), that has a signal peptide of 36 aa and a slightly shorter 513 aa long mature chain, compared to the 517 aa mature chain from *D. coronoides* [85]. Across the five sequences aligned, we observed three conserved N-glycosylation sites at positions Asn313, Asn416 and Asn531, and two putative disulfide bonds between Cys475-Cys480 and Cys479-Cys494 (in our alignment). They all show a putative chain A and B, the latter containing the catalytic triad His232-Cys233-Ser234 where the cysteine is the nucleophile responsible for the autoproteolytic cut that splits the precursor peptide into two mature chains (Figure 18).

Regarding overall venom-gland expression, *B. asper* shows the highest svPLB transcript abundance among the three species (~1.8% of the total protein transcripts), whereas svPLB expression was detected only at trace levels (<1%) in *C. d. cumanensis* and *M. mipartitus*.

##### L-Amino Acid Oxidase (svLAO)

**Figure 19:**
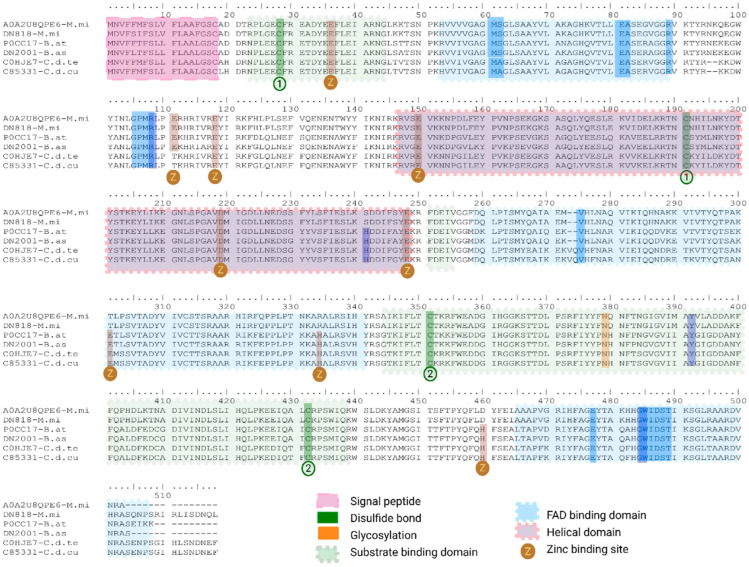
Multiple sequence alignment of svLAOs identified in this study from *M. mipartitus* DN818i9-M.mi (PX911475) with homologous sequence from *M. mipartitus* (A0A2U8QPE6), from *B. asper* DN2001-B.as (PX911476) with *B. atrox* (P0CC17) and from *C. d. cumanensis* C85331-C.d.cu (PX911477) with *C. durissus terrificus* (C0HJE7). Signal peptides, disulfide bonds and conserved domains are indicated. The figure was prepared for publication using BioRender.com.

Snake venom LAAOs (svLAOs) are flavoenzymes that catalyze the oxidative deamination of L-amino acids, producing cytotoxic hydrogen peroxide (H_2_O_2_) at the cell surface, which induces apoptosis [86]. In the transcriptome analysis of the venom glands of *B. asper*, *C. d. cumanensis* and *M. mipartitus*, only one sequence per transcriptome was annotated as svLAO. *M. mipartitus* transcript matched with *M. mipartitus* UniProtKB reviewed A0A2U8QPE6 (PPI of 99%). *B. asper* transcript matched with *B. atrox* UniProtKB reviewed P0CC17 (PPI of 98%), while *C. d. cumanensis* transcript matched with *C. d. terrificus* UniProtKB reviewed C0HJE7 (PPI of 99.8%). All these transcripts possess a signal peptide of 18 non-conserved amino acid (aa) residues, indicating that their protein products are secreted. The mature protein sequences vary in length: 498 aa residues in *M. mipartitus* and *C. d. cumanensis*, and 484 aa residues in *B. asper* (Figure 19).

Multiple sequence alignments of all three transcripts and the matched proteins revealed highly conserved active-site residues across these svLAOs, with specific differences observed at the FAD-binding site (positions 61–62 in mature chain: MA in *C. d. cumanensis* and MS in *M. mipartitus* and *B. asper*), the substrate-binding site at position 223 in the mature chain (241 with signal peptide show H in *C. d. cumanensis* and *B. asper*, S in *M. mipartitus*) (Figure 19). According to the crystal structure of L-amino acid oxidase from *Bothrops atrox*, eleven zinc-binding sites were reported by [87]. However, only nine zinc-binding sites are annotated in UniProt, which correspond to the same sites identified in *B. asper*. In contrast, eight zinc-binding sites were identified in *C. d. cumanensis* and six in *M. mipartitus*.

Expression from this family showed a strongly lineage- and method-dependent expression pattern in our datasets: it was substantial in *B. asper* (14.8 and 18.8% of the total protein TPM in Trinity and SDT31, respectively) and remained low in *C. d. cumanensis* (2.4% in Trinity and 2.5% by SDT97) and was not detected *in M. mipartitus*. LAO can be a non-trivial component of viperid venoms [88], but in many elapids, especially *Micrurus*, tends to be a minor constituent [61].

##### Hyaluronidase (HYAL)

Hyaluronidases are low-abundancy enzymes found in elapid, viperid and some colubrid venoms [66,89,90]. They are highly active degrading hyaluronic acid in the extracellular matrix (ECM), facilitating the entry of toxins into blood vessels and providing keys to the penetration of venom toxins into the neighboring tissues, thereby giving hyaluronidase its name as a “spreading factor” [91,92].

**Figure 20:**
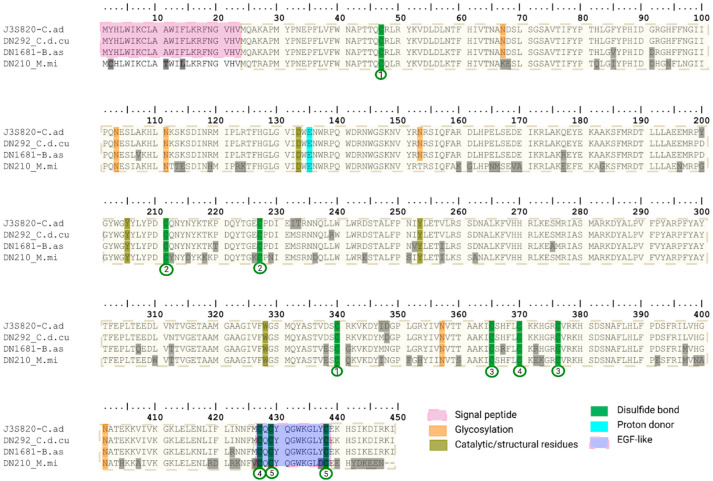
Multiple sequence alignment of hyaluronidase transcripts identified in *C. d. cumanensis* DN292_C.d.cu (PX911478), *B. asper* DN1681-B.as (PX911479), and *M. mipartitus* DN210_M.mi (PX911480) with *C. adamanteus* (J3S820). Signal peptides, disulfide bonds and conserved domains are indicated. The figure was prepared for publication using BioRender.com.

A single full length hyaluronidase transcript was identified in each of the three species analyzed (the remaining sequences were truncated). All transcripts showed their highest similarity to the reviewed hyaluronidase from *Crotalus adamanteus* (UniProtKB J3S820), with sequence identities of 98.9% for *C. d. cumanensis*, 94.4% for *B. asper*, and 83.1% for *M. mipartitus*. Vivas-Ruiz et al. (2019) [93] reported that the hyaluronidase Hyal-Ba from *B. atrox* contains six potential N-glycosylation sites (Asn67, Asn103, Asn111, Asn153, Asn357 and Asn401). In our dataset, all of these sites were conserved across the transcripts identified, except for *M. mipartitus*, in which Asn67 and Asn153 were replaced by Lys and Thr, respectively (N67K and N153T). The catalytic residue Glu135, together with other amino acids described as structurally important and highly conserved in snake hyaluronidase (Asp133, Tyr206, Tyr253 and Trp328) were also preserved in all transcripts. In addition, ten cysteine residues involved in disulfide bond formation (Cys47, Cys211, Cys227, Cys340, Cys365, Cys370, Cys376 Cys427, Cys429 and Cys438) were consistently present (Figure 20).

All viperid hyaluronidases reviewed in UniProt and listed in VenomZone (n = 8 sequences) are 449 amino acids in length, whereas those reported for *Micrurus* species (e.g., *Micrurus tener*, AOA194APD1; *Micrurus fulvius*, U3FYQ4) are shorter, with 447 amino acids. Consistent with this, the transcript from *M. mipartitus* also encoded a putative protein of 447 residues (Figure 20). Alignment analysis revealed that the *M. mipartitus* sequence displayed the greatest divergence compared to both the reference and the viperid sequences. Notably, SignalP and Phobius did not predict a signal peptide in this transcript, and two of the putative N-glycosylation sites described in *C. adamanteus* were absent (Figure 20). Interestingly, the *Micrurus* sequences (including *M. mipartitus*) encode a predicted signal peptide beginning with MCH, in contrast to the MYH motif typical of viperid hyaluronidases. These differences raise questions regarding the processing, secretion and stability of the *M. mipartitus* transcript. Experimental validation will be required to determine whether it encodes a functional hyaluronidase. Hyaluronidases represent less than 1% of the total protein expression in all assemblies.

##### Vespryn/Ohanin

**Figure 21:**
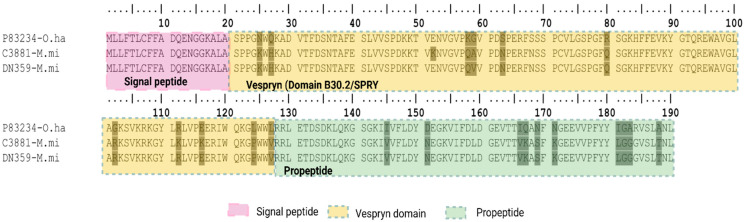
Multiple sequence alignment of the two vespryn/ohanin transcripts identified in *M. mipartitus* C3881-M.mi (PX911481) and DN359-M.mi (PX911482) with *Ophiophagus hannah* (P83234). Polymorphic sites related to P83234 are shaded in grey. Signal peptides, disulfide bonds and conserved domains are indicated. The figure was prepared for publication using BioRender.com.

Snake-venom vespryns/ohanins are secreted, non-enzymatic proteins reported predominantly from elapid venoms, with only rare occurrences in crotalines where they are typically of low abundance (<0.5%) [89]. In this study, only in *Micrurus mipartitus* venom-gland transcriptome we identified two transcripts (C3881-M.mi, DN359-M.mi); no vespryns were detected in *Bothrops asper* or *Crotalus durissus cumanensis*. BLAST against UniProt (reviewed) showed 89.5% identity to ohanin from *Ophiophagus hannah* (UniProtKB P83234). When we analyzed the signal peptide prediction of these transcripts, the SignalP v6.0 prediction probability was below the desired threshold (*p* ≥ 0.90). When we used Phobius, the software predicted a signal peptide of 20 aa, showing consistency with the curated UniProtKB P83234 entry from *O. hannah* which reports a 20 aa signal peptide supported by manual assertion (Figure 21). Vespryn/ohanin tends to be a minor/trace component across lineages when present, as those found here in *M. mipartitus* that showed <1% contribution to the total protein expression.

##### Natriuretic Peptide Family (VNPs)

**Figure 22:**
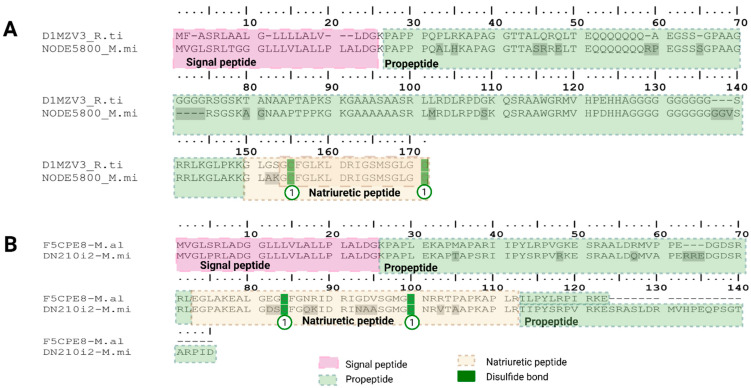
Sequence alignment of natriuretic peptides identified in (**A**). *M. mipartitus* NODE5800-M.mi (PX911483) with homologous sequences from *Rhabdophis tigrinus tigrinus* (D1MZV3); and (**B**) *M. mipartitus* DN210i2-M.mi (PX931517) with *M. altirostris* (F5CPE8). Polymorphic residues relative to their respective hits are highlighted in grey. Signal peptides, disulfide bonds and conserved domains are indicated. The figure was prepared for publication using BioRender.com.

Natriuretic peptides in snake venoms are vasoactive proteins that mimic endogenous mammalian natriuretic hormones. Functionally, they induce vasodilation, natriuresis, and diuresis, thereby contributing to a rapid and sustained drop in blood pressure in envenomed prey [94]. In *Micrurus mipartitus*, two distinct natriuretic peptide (NP) transcripts were identified, both containing a 25 aa signal peptide. One copy matched the colubrid *Rhabdophis tigrinus tigrinus* sequence (D1MZV3 UniProtKB reviewed; PPI of 79%) and displayed the conserved mammalian natriuretic peptide motif CFGXXXDRIXXXXGLGC, forming the canonical 17 residue ring stabilized by a disulfide bond (Figure 22A). This structural element is essential for receptor binding, and amino acid variability within the ring confers receptor specificity [94]. This sequence carried an additional five amino acids (GLAKG) upstream of the conserved cysteine and terminated in the typical GLGC. The second copy showed homology to the natriuretic peptide from *Micrurus altirostris* (F5CPE8 UniProtKB reviewed; PPI of 81.3%) but diverged from this consensus, containing ten residues preceding the conserved cysteine, ending in GMGC instead of GLGC, and extended by twelve extra residues at the C-terminal region (Figure 22B). Interestingly, similar sequence extensions have also been reported in elapids such as *Naja atra*, where a novel NP (Na–NP) of 45 amino acids was purified and characterized, with its full-length cDNA encoding a 165 amino acid precursor [95].

Snake venom NPs share the same 17-residue ring structure as mammalian NPs and differ in the length and sequence of their N- and C-terminal tails [94], a pattern reflected in the structural variation observed between the two *M. mipartitus* copies.

By similarity, the canonical NP of *M. mipartitus* may display vasorelaxant and diuretic activities, as experimentally demonstrated for truncated CNPs from the viperid *Trimeresurus flavoviridis* [96]. Likewise, the non-canonical NP may induce rapid relaxation of phenylephrine-precontracted rat aortic strips, act through stimulation of cGMP production via NPR1 and/or NPR2 activation, and exert potent hypotensive effects, in a dose-dependent form, in agreement with the functional properties reported for the extended Na–NP from *Naja atra* (UniProtKB D9IX97; [95]). These functional inferences highlight how structural diversification in *M. mipartitus* NPs could broaden the pharmacological spectrum of its venom. Experimental validation will therefore be essential to confirm the biological activities of the *M. mipartitus* natriuretic peptides. As observed in the BPP section, expression of NPs in viperids mainly corresponds with BPP-CNP precursors, while in elapids, single NP transcripts can be found, usually a minor protein class, as seen in *M. mipartitus* were VNP expression was <1% of the total protein family expression.

##### Coagulation Factor X (CFX)

In vertebrates, Factor X (F10) is a central component of the coagulation cascade: it is a vitamin-K-dependent, N-glycosylated serine protease that, within the prothrombinase complex and in the presence of cofactor Va, Ca^2+^ and phospholipids, converts prothrombin to thrombin [97].

**Figure 23:**
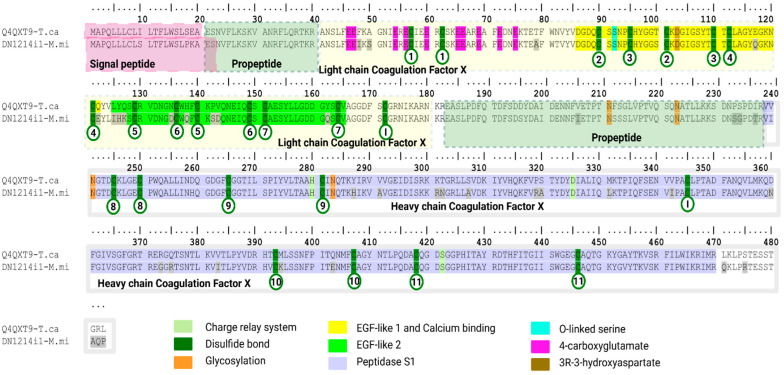
Multiple sequence alignment of CFX transcripts from *M. mipartitus* DN1214i1-M.mi (PX911484) with homologous sequence from *Tropidechis carinatus* (Q4QXT9). Signal peptides, disulfide bonds and conserved domains are indicated. The figure was prepared for publication using BioRender.com.

We identified one FX-like transcript from *M. mipartitus* (DN1214i1-M.mi), showing the highest similarity to *Tropidechis carinatus* Factor X (Q4QXT9 UniProtKB reviewed sequence; PPI of 88.6%), a 483 aa long precursor chain with a 19 aa signal peptide, while a predicted cleavage signal for DN1214i1-M.mi occurs at position 22 (Figure 23). The propeptides, PP-Gla (position 21–40) carry γ-carboxylatable Glu residues and the activation peptide (183–238) is cleaved by FVIIa/TF (extrinsic) or FIXa/FVIIIa (intrinsic) pathways [97]. The Factor X light chain (aa 41–180) has (1) a Gla domain with multiple Gla residues and one O-linked Ser, (2) an EGF-like 1 domain (aa 86–122) with Ca^2+^-binding and (3) an EGF-like 2 domain (aa 125–165). The heavy chain (aa 183–483) is composed of a peptidase S1 domain (aa 239–470) (trypsin-like) that contains a catalytic charge-relay system His280, Asp325, with the nucleophilic Ser422 in the DSGG motif [98]. This transcript represents 0% of the total proteins recovered, whereas in Australian-type elapids (*Pseudonaja*) prothrombinase systems can dominate transcript expression [99].

## 3. Discussion

### 3.1. Assembler Performance and Methodological Implications

By integrating diverse multi-assembler transcriptomic analysis, we achieved a high-resolution map of the protein repertoire, revealing venom gland complexity beyond dominant protein groups from Viperidae and Elapidae. Our findings highlight a significant diversity of low-abundance enzymatic and non-enzymatic proteins families, which are often overlooked in standard assemblies [100,101]. These minor components contribute to the functional versatility of the venom and provide crucial insights into evolutionary adaptations [102]. The combined analysis of assembly quality metrics and curated proteins annotation demonstrated that assembler choice has an impact not only on transcriptome completeness and redundancy, but also on the inferred venom composition and expression landscape [100,103]. Although all assemblies were subjected to the same downstream annotation and redundancy-filtering pipeline, each assembler introduced distinct and biologically meaningful biases over each protein family recovery and relative expression. Trinity consistently maximized transcript and protein-family diversity, recovering a higher number of unique and exclusive protein transcripts, particularly low-abundance and rare copies that are typically underrepresented. This sensitivity is advantageous for venoms where multigene protein families and copy variation are central to functional diversity and evolution [100]. SPAdes produced more compact assemblies with lower redundancy while still contributing a substantial fraction of exclusive protein transcripts, especially in viperids, indicating that reduced duplication does not necessarily compromise biological resolution. Instead, SPAdes complements Trinity by resolving transcripts that may be collapsed or fragmented in redundancy-rich assemblies [103]. SOAPdenovo-Trans performance was highly k-mer dependent. SDT31 (k = 31) showed increased sensitivity, whereas SDT97 (k = 97), recognized fewer effective reads and imposing stringent coverage requirements, exhibited marked fragmentation, loss of the protein families, and reduced copy diversity, particularly in *Micrurus mipartitus*, therefore generating bias toward dominant toxins. This performance is consistent with previous observations in the de Brujin graph-based transcriptome assemblers, where assembly quality is deeply influenced by k-mer length and no single k-mer value yields optimal results across heterogeneous transcriptome; instead, combining assemblies generated with different k-mers improves sensitivity and transcript recovery [104,105].

Importantly, integration of multiple assemblers, combined with thorough manual curation of low abundance enzymatic and non-enzymatic protein annotations, was essential to validate the recovered protein diversity and to confidently distinguish true gene copies from assembly artefacts or chimeric transcripts [106]. This combined strategy maximized the detection of previously unreported protein variants, providing a robust framework for studying venom evolution driven by gene duplication, copy retention, and differential expression.

### 3.2. Transcript Variants Diversity and Evolutionary Perspectives

The interspecific comparison revealed intra-family transcript diversity and marked lineage-skewed presence/absence and transcript variant patterns between the viperids (*B. asper* and *C. d. cumanensis*) and the elapid *M. mipartitus.* Notably, *M. mipartitus* showed the highest overall transcripts variant richness across all family categories (secondary: 20 vs. 14–15 in viperids; minor: 19 vs. 8; rare: 9 vs. 5) (see Table 2).

First, Kunitz-type inhibitors represent a widespread venom gene family across advanced snakes, with diversification patterns consistent with recurrent duplication and lineage-specific retention [107,108]. In this analysis, Kunitz type protease inhibitor (KSPI) family showed the most pronounced disparity: in addition to the long (two tandem Kunitz domains), and multidomain Kunitz form shared with viperids, *M. mipartitus* contained multiple Kunitz–WAP fusions (Ku-WAP) (*n* = 5) a rare architecture originally described as a Kunitz/WAP domain fusion and interpreted as lineage-specific domain shuffling/duplication rather than a universal venom component [10], and a short (single Kunitz) transcript variant, (*n* = 1), which were not detected in the viperid transcriptomes. Notably, the presence of Kunitz type inhibitors in *M. mipartitus* has been previously reported by [28] and our dataset expands this observation by resolving additional transcript level diversity within this family.

VEGF-like transcripts also differed by lineage. Both viperids and *M. mipartitus* present VEGF-A and VEGF-C transcripts, but *M. mipartitus* showed a higher representation of VEGF-A ΔHBD variants (*n* = 2 vs. 1 in viperids), whereas, in both viperids we identified two VEGF-A transcripts (*n* = 1 each) homologous to the TfVEGF122 variant described for *Protobothrops flavoviridis* (UniProt P67860-3), which is characterized by a large internal deletion corresponding to residues 143–210. This truncated architecture contrasts with the full-length VEGF-A forms and supports the occurrence of structurally distinct VEGF-A transcript variant in viperid venoms.

Snake venom VEGFs (often referred to as VEGF-F/svVEGF) have been proposed to represent venom-specific VEGF-like molecules distinct from the endogenous VEGF-A system required for physiological angiogenesis, rather than simple splice variants of VEGF-A. Indeed, biochemical and genetic evidence indicates that venom VEGFs can display receptor-binding and functional profiles that diverge from vertebrate VEGF-A isoforms, supporting lineage and venom gland specific recruitment of VEGF-like toxins [33]. In this context, the lack of detectable VEGF-F transcripts in *M. mipartitus* suggests that this elapid may rely on alternative VEGF-like repertoires (e.g., VEGF-A derived transcript such as ΔHBD variants and/or VEGF-C-like transcripts) to achieve vascular effects during envenoming. This interpretation is consistent with comparative transcriptomic evidence indicating that VEGF-F sequences are not broadly represented across elapids, supporting a lineage-specific distribution of venom-type VEGFs [109].

In contrast ENPP-type phosphodiesterases showed an opposite pattern. Genomic and transcriptomic evidence indicates that svPDE represents a protein family co-opted by the venom gland from an ancestral ENPP3 locus by alternative splicing, which replaces the cytoplasmic/transmembrane region with an N-terminal signal peptide, yielding a soluble venom form [110]. In *M. mipartitus*, we did not detect ENPP3-like transcripts and observed a distinct expression split among ENPP type PDE, suggesting an alternative elapid strategy to retain soluble ENPP-type PDE activity without ENPP3. These findings suggest great selective pressure in order to maintain soluble PDEs in snake venom. In viperids, we additionally identified transcript variants with and without SMB1 domains. To date, there is no experimental evidence demonstrating functional differences attributable to SMB1 loss; however, because SMB modules are generally considered stabilizing/interaction cassettes, their absence may affect stability, secretion, and/or processing, but this remains to be tested [110]. Overall, these patterns highlight that gene expansion and/ or retention are uneven across protein families and lineages.

We identified multiple lysosomal acid lipase (LIPA)-like transcript variant in *M. mipartitus* (*n* = 6), supporting lineage specific enrichment of this low-abundance component within our dataset. LIPA-like transcripts have previously been reported as ‘novel’ venom-gland products in viperid transcriptomes (e.g., *Echis coloratus*) [29] and, importantly, were also detected at both the transcriptome and venom-proteome levels in an elapid (*Micrurus altirostris*), lending support to their recruitment into venom systems [36]. Proteotranscriptomic work has further argued that this component represents a true venom protein and introduced the term svLIPA (snake venom acid lipase) [108]. Given that canonical lysosomal acid lipase (LIPA/LAL) functions as a cholesteryl-ester and triglyceride hydrolase in lysosomal lipid catabolism [111], the pathophysiological role of svLIPA in envenoming remains unresolved [36]; nevertheless, its repeated detection across lineages and the transcript diversity observed here motivate targeted validation (venom proteomics and activity assays) to clarify whether it contributes to lipid/membrane remodeling, tissue damage, or other ancillary venom effects.

### 3.3. High Diversity in Minor and Rare Protein Classes

Differences were more pronounced in minor and rare protein classes. *M. mipartitus* exhibited markedly higher transcript counts (minor: n = 19; rare: n = 9) than viperids (minor: 8 and 8; rare: 5 and 5 in *C. d. cumanensis* and *B. asper*, respectively). Across families, transcript-level variation comprises residue substitutions, short indels, signal-peptide differences, and changes in predicted domain architecture. (e.g., CTL-STRICT variants, CRISP, ficolins, LIPA). Canonical C-type lectins (CTLs) differed markedly among the three species, consistent with lineage-specific variation in the CRD tripeptide motif that underlies carbohydrate specificity [11,112]. Because QPD is generally associated with galactose binding and EPN with mannose binding [113], the QPD-bearing CTLs recovered in *Bothrops asper* are consistent with previously described viperid galactose-binding lectins [114,115]. Additional variants have been reported with unknown specificity [113]. Notably, *B. asper* contained two transcripts encoding an identical mature CTL chain but differing in predicted signal-peptide length, suggesting conserved protein cores with variable secretion signals. In contrast, no galactose-binding CTLs were detected in *Micrurus mipartitus*, despite reports of both mannose- and galactose-binding CTLs across Elapidae (VenomZone, ExPASy), and no canonical CTLs were recovered in *Crotalus durissus cumanensis*. Structural data from BJcuL a C-type lectin from *B. jararacussu* indicate that galactose-binding CTLs can form multimeric assemblies and bind ligands beyond carbohydrates, including aminoglycosides [54] and affects the ubiquitin proteasome system acting as a deubiquitinase activator [116]. These findings suggest that the viperid CTLs identified here may have broader functional potential.

Ficolins are soluble pattern-recognition receptors that bind acetylated glycans and other PAMPs through their fibrinogen-like (FBG) domain. This FBG domain promotes opsonization and lectin-pathway complement activation via MASP recruitment [117,118]. In our dataset, ficolin transcripts were detected exclusively in *Micrurus mipartitus* and were not recovered in the viperids, representing to our knowledge the first report of ficolins in an elapid species, and suggesting a lineage-specific recruitment of a pattern-recognition molecule into the venom-gland repertoire. Structural work indicates that ligand specificity resides in the FBG domain, whereas the collagen-like region mediates oligomerization and MASP docking [119,120]. This finding expands the spectrum of immune-related proteins potentially co-opted into venoms and motivates targeted functional validation to assess whether these ficolin-like products contribute to antimicrobial or immunomodulatory effects and may have bioprospecting value [117,118].

Bradykinin-potentiating peptides (BPPs) are modular venom peptides that inhibit ACE receptor and enhance bradykinin activity. This pharmacological principle ultimately inspired captopril development [69,72,74]. In our dataset, BPP-related diversity was strongly lineage dependent: *Micrurus mipartitus* lacked detectable BPPs, whereas viperids retained BPP modules with clear structural variation. In *Bothrops asper*, BPP transcripts showed differences consistent with gains/losses of specific peptide modules, a pattern best explained by rearrangements within the modular N-terminal exon of the BPP/CNP precursor rather than independent gene duplications [121]. Notably, *C. d. cumanensis* was the only species in this study encoding the BIP peptide (TPPAGPDVGPR-OH), originally characterized from viperid venoms and subsequently annotated in multiple crotaline lineages [122]. Collectively, these results emphasize that BPP repertoires vary primarily through modular architecture and lineage-specific retention, with *Crotalus* uniquely retaining BIP in our sampling and *M. mipartitus* lacking this peptide class altogether [121,122].

Venom natriuretic peptides (VNP/NPs) were recovered only in the elapid in our dataset (Table 2); however, natriuretic toxins occur across advanced snakes and their apparent presence/absence can reflect differences in precursor architecture and annotation granularity, as many viperids encode natriuretic peptide domains within BPP/CNP precursors [123,124], while standalone NPs have been purified and characterized from elapid venoms (e.g., *Naja atra*) [95].

NGF can be viewed as a basal venom-associated family, i.e., a toxin/toxin-like component likely recruited early and broadly retained across major snake lineages rather than restricted to a single clade [125]. Consistent with this expectation, NGF transcripts were recovered in all three focal taxa in our dataset (Table 2), despite typically low copy number and low expression Snake venom NGFs have been reported to carry an N-terminal histidine rich motif within the mature chain [e.g., H(X)2H(X)2GX], though this pattern is not universal across taxa [126]. In our dataset, the *M. mipartitus* transcript DN359 retains the canonical pattern (HPVHNQG, matching H-X_2_-H-X_2_-G), whereas NODE10366 does not: its N-terminal segment (HPVYDL) lacks the second histidine (replaced by Tyr) and the glycine (one-residue deletion), thus deviating from the consensus. Similar exceptions have been noted for some elapid NGFs in UniProt reviewed entries (e.g., *Tropidechis carinatus* Q3HXX4; *Demansia vestigiata* A6MFL5–A6MFL7; UniProtKB, reviewed). Prior surveys indicate that sNGFs span broad ranges in acidic (Asp, Glu) and basic (Arg, Lys, His) residues, with many sNGF falling into the basic isoelectric point (pI) range; however, *Bothrops cotiara*, *Walterinnesia aegyptia* and *Pseudechis australis* show acidic sNGFs (acidic pI values), indicating that sNGFs occur in both basic and acidic forms [126]. Using a protein isoelectric point calculator http://isoelectric.org/ (accessed on 18 December 2025) on the mature chains, we found basic sNGFs in *B. asper* (pI = 7.250) and *C. d. cumanensis* (pI = 7.512), and acidic sNGFs in *M. mipartitus* (DN359, pI = 5.758; NODE10366, pI = 6.095). These data support classifying our sNGFs into basic (*B. asper, C. d. cumanensis*) and acidic (*M. mipartitus*) variants under the IPC model adopted here.

Finally, similar broadly retained (“basal”) patterns in our three species were also observed for other low-abundance families such as phospholipase B (PLB), L-amino-acid oxidase (LAO) and hyaluronidase (HYAL) (Table 2), supporting the idea that a subset of venom-gland families is conserved across both viperids and elapids even when they are not dominant contributors to venom composition [125].

Importantly, rigorous manual curation of protein annotations, supported by UniProt hits, and explicit filtering of likely chimeric sequences were required to validate true transcript variants and avoid artefactual inflation of copy diversity. While no formal evolutionary analyses were conducted, the observed lineage- and family-specific transcript variant patterns (particularly the expanded KSPI repertoire in the elapid lineage) provide a robust empirical basis to discuss differential recruitment and diversification of protein copies across snake families.

### 3.4. Innovative Potential of the Protein Arsenal

Comprehensive protein cataloguing is of strategic value for innovation. This is of particular interest for low-abundance families and isoforms as it expands the searchable space of bioactives for translation, since venoms have repeatedly yielded drug leads and approved therapeutics (e.g., ACE inhibition inspired by BPPs/captopril; ion-channel modulators such as ziconotide) and continue to provide high-specificity ligands for receptors, enzymes, and ion channels [127]. The innovation value of an expanded venom repertoire lies in (i) new molecular scaffolds (including lineage-specific isoforms and domain architectures), (ii) target selectivity suited for precision pharmacology and (iii) opportunities for engineering and delivery, where venom peptides can be adapted as targeting moieties or payloads in bioconjugates/nanoparticle systems [128].

In this context, we identify four transcripts encoding NGF. In humans, insufficient NGF availability or dysregulated NGF signaling has been associated to several neurodegenerative conditions such as Alzheimer’s Disease (AD), Parkinson’s Disease (PD), depression and hypoxic ischemic perinatal brain injury [126]. Snake venom NGF (sNGF) is structurally and functionally similar to human NGF (hNGF), activating the same canonical pathways via trkA and p75 receptors [129,130] and has been proposed to offer better bioavailability, efficacy and stability compared to other sources [126]. Collectively, the NGF isoforms recovered here represent tractable candidates for downstream functional validation and may provide a starting point for exploring NGF-based therapeutic development.

In addition, we identified three cystatin encoding transcript (one in each analyzed specie). Snake venom cystatins belong to the cystatin-like superfamily of cysteine protease inhibitors, which target cathepsins B, L, H and S [131,132]. Beyond their canonical anti cathepsin activity, cystatin derived peptides have shown translational promise against *Trypanosoma cruzi*. Díaz et al. (2025) [133], reported AsCystatin and four derived peptides with inhibitory activity against cruzain, with in vitro trypanocidal effect. Importantly, our cystatin sequences retain the conserved asparagine in the backside loop, a determinant required for legumain inhibition [134]. Given that legumain has been increasingly implicated in the onset and progression of cardiovascular diseases, including carotid atherosclerosis, pulmonary hypertension, coronary artery disease, peripheral arterial disease, aortic aneurysms and dissection [135]. The cystatins recovered here may represent dual-interest candidates, both as antiparasitic scaffolds (via cruzain targeting) and as starting points for exploring legumain-modulatory strategies in cardiovascular pathology.

Finally, we identified two 5′-nucleotidaseencoding transcripts in each viperid (*B. asper* and *C. d. cumanensis*), supporting the presence of this enzyme as a conserved venom associated component. Given that serum 5-nucleotidase has been reported as an effective biomarker for assessing snakebite severity and predicting outcomes showing a strong correlation with clinical severity scores [136], these viperid nucleotidases represent attractive candidates for translational development. In particular, they could motivate the design of rapid detection reagents (e.g., binding molecules/affinity probes) to enable near-point-of-care quantification and improve early severity stratification alongside clinical assessment.

To summarize, our transcriptomic and analyses identify potentially useful proteins for innovative therapies for neurodegenerative, cardiovascular and specific parasitic diseases, in addition to improving diagnosis of snake venom accidents in their countries of origin. Importantly, recent work emphasizes that low-abundance components are systematically under-characterized but represent a major reservoir for discovery; integrating multi-assembler transcriptomics with stringent annotation/curation directly addresses this bottleneck and increases confidence in prioritizing candidates for functional validation and bioprospecting [137].

## 4. Conclusions

This study provides a comprehensive transcriptomic analysis of the venom glands of three Colombian medically significant snake species: *M. mipartitus*, *B. asper* and *C. d. cumanensis.* Our results reveal a remarkable diversity of venom transcript variants, which include both enzymatic and non-enzymatic proteins, many of which are low abundance components often underrepresented in traditional venom studies. The integration of multiple transcript assemblers, combined with rigorous manual curation, was essential for identifying transcript variants previously reported in other snake species, but not in the venom gland of these Colombian species, thus highlighting the power of multi-assembler strategies in uncovering hidden protein diversity.

The comparison between species revealed distinct patterns in venom transcript variants, with *M. mipartitus* exhibiting a higher transcript variant richness across all protein categories compared to the viperids. For example, *M. mipartitus* showed a greater diversity of Kunitz-type protease inhibitors, VEGF-like factors, and phosphodiesterases, which are crucial for understanding lineage-specific adaptations in venom composition. In contrast, *B. asper* and *C. d. cumanensis* displayed more conserved venom profiles, with fewer transcript variants and a more limited repertoire of rare proteins.

Importantly, while this study reveals an array of novel transcripts variants, functional validation of these transcripts is essential to confirm their role in venom activity and their potential as therapeutic or diagnostic targets. Further investigation into the biological activities of these newly identified variants will be crucial for understanding their specific contributions to envenomation and their evolutionary significance.

In summary, we observed that Trinity and SPAdes assemblers may be more suitable to maximize transcript and protein-family diversity with lower redundancy from snake venoms. Despite this, our work emphasizes the importance of integrating multiple assembly tools and rigorous manual curation to accurately capture the complexity of venom gland transcriptomes. The high diversity of venom components identified across species presented here offers a reliable basis for subsequent experimental validation, drug development and the design of diagnostic tools for snakebite envenomation.

## 5. Materials and Methods

### 5.1. Venom Gland Tissue and RNA Extraction

Biological samples were obtained in agreement with the contract for access to genetic resources and their derivative products N°370 and 126 (addendum #14), approved by the Ministry of Environment and Sustainable Development of Colombia. Venom gland tissues were provided by the serpentarium tissue bank of the Toxinology, Therapeutic and Food Alternatives research group at the University of Antioquia, Colombia. Samples were collected from three venomous snake species: *Bothrops asper* (female, 1.15 m, voucher N° 4457, Barbosa-Antioquia region), *Crotalus durissus cumanensis* (male 1.21 m, voucher N° 4446, unknown region), and *Micrurus mipartitus* (female, 88.2 mm, voucher N° 4654, Jericó-Antioquia region). Venom glands were excised immediately after natural death, immersed in RNA later (Thermo Fisher Scientific), and stored at −80 °C until RNA extraction. Total RNA was isolated and quality-checked at the Centro Nacional de Secuenciación Genómica (CNSG), University of Antioquia, following protocols described in Saldarriaga et al., 2024 [28].

### 5.2. Transcriptome Sequencing and De Novo Assembly

RNA libraries were prepared using Illumina TruSeq Stranded mRNA kits and sequenced (150 bp paired end) on a NovaSeq 6000 platform. Raw reads were filtered using CUTADAPT v3.5, removing adapter sequences, reads with Phred quality < Q30, and reads shorter than 70 bp. Cleaned reads were assembled de novo with four independent assemblers to maximize transcript recovery: Trinity v2.13.2 (GitHub—trinityrnaseq/trinityrnaseq: Trinity RNA-Seq de novo transcriptome assembly; [138]), rnaSPAdes v3.14.1 (k = 31) [139] and SOAPdenovo-Trans v1.03 at two k-mer sizes (k = 31 and k = 97) [140]. Default parameters were used unless specified. Assembly completeness and contiguity were evaluated with BUSCO v5.8.0 (eukaryota_odb10 dataset; [141]) and TransDecoder v5.5.0, both executed through the Galaxy platform (usegalaxy.org; accessed December 2025). Assemblies were then merged and clustered with CD-HIT-EST (v4.8.1, 95% identity) to remove redundancy and generate a non-redundant meta-assembly for each species. Basic transcriptome statistics (N50, GC content, mean contig length) were computed with custom Python (v3.11) scripts developed by the Centro Nacional de Secuenciación Genómica of the University of Antioquia.

### 5.3. Quantification of Gene Expression

Cleaned reads were pseudo-aligned to each species’ non-redundant transcript set using Kallisto v0.44.0. Transcript abundance was estimated as transcripts per million (TPM). The resulting TPM matrices (TPMsfbvag) were used to quantify each protein-family expression by summing TPM values across all curated protein transcripts assigned to each family. Family-level relative expression was calculated as the percentage of total protein TPM per assembly and species. Expression summaries and plots were generated in R (v4.4.1) using tidyverse/ggplot2, and correlation analyses between assembly metrics and expression patterns were performed using Pearson’s correlation coefficient.

### 5.4. Functional Annotation and Manual Sequence Curation

Protein transcripts were detected and separated by ToxCodAn v1.0 package, available at https://github.com/pedronachtigall/ToxCodAn ([16], package downloaded and installed on 5 May 2023). ToxCodAn is a computational tool designed to identify and annotate toxin/protein transcripts from de novo assembled transcriptomes. This pipeline uses BLAST and trained generalized Hidden Markov Models (gHMM) to identify protein CDSs. Primary structures of toxins identified by ToxCodAn were submitted to analysis on SignalP-6.0 server https://services.healthtech.dtu.dk/services/SignalP-6.0/ (accessed on 1 October–18 December 2025), applying a conservative probability threshold (*p* ≥ 0.90) [17,142] and confirmed by Phobius webserver to minimize false positives [18] in order to predict the cleavage sites of signal peptide. SignalP-6.0 applies conservative criteria and may fail to detect signal peptides affected by polymorphism in the N or H regions. Therefore, transcripts showing reduced prediction scores were still retained for comparative analyses when they displayed strong sequence homology and conserved domain architecture, including characteristic motif (e.g., conserved cysteine patterns, catalytic/functional features), and were supported by Phobius predictions. Mature sequences were also submitted to homology search in UniProtKB/Swiss-Prot https://www.uniprot.org/ (accessed on 1 October–18 December 2025), venom zone database (accessed on 26 December 2025, https://venomzone.expasy.org/). To validate protein transcripts with an unreviewed highest hit, 3D structure homology models were constructed. The models are contrasted with expected structure for the corresponding to each protein family by identification of putative active sites in the mature chain. http://www.sbg.bio.ic.ac.uk/phyre2/html/page.cgi?id=index (accessed on 1 October–18 December 2025). Domain architecture validation. Conserved motifs and domains were confirmed using NCBI CDD (Conserved Domain Database https://www.ncbi.nlm.nih.gov/Structure/cdd/wrpsb.cgi, accessed on 1 October–18 December 2025) and InterProScan (https://www.ebi.ac.uk/interpro/about/interproscan/, accessed on 1 October–18 December 2025). All validated sequences were aligned with MAFFT v7.490 [143] and visually inspected in BioEdit to confirm motif conservation across transcript variants.

## Figures and Tables

**Table 1:** Assembly quality by each assembler used in the venom gland transcriptome of A. *Bothrops asper*, B. *Crotalus durissus cumanensis* and C. *Micrurus mipartitus*.

**A. *Bothrops asper***				
	**Trinity**	**SPAdes**	**SDT_K31**	**SDT_K97**
Assembly_length_ bp	80,293,964	47,346,633	63,215,732	35,268,235
Scaffold_count	80,782	50,885	154,110	42,208
Scaffold_average_bp	993	930	410	831
Largest_scaffold_bp	14,136	12,410	14,140	11,723
N50_bp	2102	1751	1118	1460
GC_ratio	42.37	42.57	41.71	42.34
N_count	0	540	489,251	278,852
N_ratio	0	0	0.77	0.79
**B. *Crotalus durissus cumanensis***				
	**Trinity**	**SPAdes**	**STD_K31**	**SDT_K97**
Assembly_length_ bp	76,577, 626	43,492,464	62,136,982	29,962,733
Scaffold_count	83,292	51,478	163,820	38,930
Scaffold_average_bp	919	844	379	769
Largest_scaffold_bp	14,801	11,072	12,566	11,521
N50_bp	1781	1453	884	1184
GC_ratio	43.3	43.2	42.28	43.14
N_count	0	510	338,217	145,482
N_ratio	0	0	0.54	0.49
**C. *Micrurus mipartitus***				
	**Trinity**	**SPAdes**	**STD_K31**	**SDT_K97**
Assembly_length_ bp	75,874,010	37,481,895	67,415,284	1,081,409
Scaffold_count	84,949	53,604	216,459	1691
Scaffold_average_bp	893	699	311	639
Largest_scaffold_bp	12,512	9848	12,063	5499
N50_bp	1828	1085	772	761
GC_ratio	40.61	40.82	39.86	41.8
N_count	0	0	1,507,731	20,570
N_ratio	0	0	2.24	1.9

**Table 2:** Curated putative protein variants by transcript count categories (secondary, minor, rare) across species.

Category/ Transcripts Number	Family	Isoform Type		*C.d.cu*	*B. as*	*M. mi*	Observations
Secondary	KSPI	Short KSPI		0	0	1	Residue substitutions in SP and mature chain, indel
		Long KSPI	Insertion	1	1	1	
			Deletion	1	1	0	
		Ku-WAP fusin		0	0	5	
		Multidomain		1	1	1	
Secondary	VEGF	VEGF-A	HBD	1	1	1	Deletion and residue substitutions
			Δ HBD	1	1	2	
			VEGF122	1	1	0	
		VEGF-C		1	1	1	Residue substitutions
		VEGF-F		1	2	0	Deletion and residue substitutions
Secondary	PDE	ENPP3-like	SMB1	1	2	0	Deletion and residue substitutions
			Δ SMB1	1	1	0	
		ENPP4-like		1	2	1	Residue substitutions
		ENPP6-like		2	0	1	Indel in SP and residue substitution
Secondary	LIPA			1	1	6	Residue substitution
Total				14	15	20	
Minor	NGF			1	1	2	Residue substitution and indel
Minor	CTL strict	WIGL/QPD/WND		0	2	0	Variable SP length
		WIGL/EPN/WND		0	0	4	Residue substitution in SP and three mature variants
Minor	Waprin	Double Waprin domain		1	1	1	Residue substitution in SP and mature chain
		Single Waprin domain		1	1	1	
Minor	CRISP			2	0	4	Residue substitution in SP and mature chain
Minor	Ficolin			0	0	5	Three variants in SP and mature chain substitution residues
Minor	BPP	BPP/BIP		1	0	0	Two variants in SP, indels and specific domains
		Δ BPP+ poly His-poly Gly peptide		0	3	0	
Minor	AChE	T isoform		2	0	0	Three variants in SP, indels and specific domains
		Δ tetramerization site		0	0	1	
		S isoform		0	0	1	
Total				8	8	19	
Rare	Cystatins			1	1	1	Residue substitution in SP and mature chain
Rare	PLB			1	1	1	Residue substitution in mature chain
Rare	LAO			1	1	1	Residue substitution in SP and mature chain
Rare	HYAL			1	1	1	Residue substitution in SP and mature chain
Rare				0	0	1	
Rare	NUC			1	1	0	
Rare	VNP			0	0	2	
Rare	CFX			0	0	1	
Rare	VCO3			0	0	1	
Rare	VDP4			1	0	0	
Total				5	5	9	

## Data Availability

The original data presented in this study are openly available in GenBank database, reference number PX911394–PX911484 and PX9315.
